# Highly efficient fermentation of 5-keto-d-fructose with *Gluconobacter oxydans* at different scales

**DOI:** 10.1186/s12934-022-01980-5

**Published:** 2022-12-10

**Authors:** Svenja Battling, Tobias Engel, Elena Herweg, Paul-Joachim Niehoff, Matthias Pesch, Theresa Scholand, Marie Schöpping, Nina Sonntag, Jochen Büchs

**Affiliations:** AVT-Chair for Biochemical Engineering, RWTH Aachen University, Forckenbeckstraße 51, 52074 Aachen, Germany

**Keywords:** *Gluconobacter oxydans*, 5-Ketofructose, Fructose dehydrogenase, Extended batch fermentation, Scale-up

## Abstract

**Background:**

The global market for sweeteners is increasing, and the food industry is constantly looking for new low-caloric sweeteners. The natural sweetener 5-keto-d-fructose is one such candidate. 5-Keto-d-fructose has a similar sweet taste quality as fructose. Developing a highly efficient 5-keto-d-fructose production process is key to being competitive with established sweeteners. Hence, the 5-keto-d-fructose production process was optimised regarding titre, yield, and productivity.

**Results:**

For production of 5-keto-d-fructose with *G. oxydans* 621H Δ*hsdR* pBBR1-p264-*fdhSCL*-ST an extended-batch fermentation was conducted. During fructose feeding, a decreasing respiratory activity occurred, despite sufficient carbon supply. Oxygen and second substrate limitation could be excluded as reasons for the decreasing respiration. It was demonstrated that a short period of oxygen limitation has no significant influence on 5-keto-d-fructose production, showing the robustness of this process. Increasing the medium concentration increased initial biomass formation. Applying a fructose feeding solution with a concentration of approx. 1200 g/L, a titre of 545 g/L 5-keto-d-fructose was reached. The yield was with 0.98 g_5-keto-d-fructose_/g_fructose_ close to the theoretical maximum. A 1200 g/L fructose solution has a viscosity of 450 mPa∙s at a temperature of 55 °C. Hence, the solution itself and the whole peripheral feeding system need to be heated, to apply such a highly concentrated feeding solution. Thermal treatment of highly concentrated fructose solutions led to the formation of 5-hydroxymethylfurfural, which inhibited the 5-keto-d-fructose production. Therefore, fructose solutions were only heated to about 100 °C for approx. 10 min. An alternative feeding strategy was investigated using solid fructose cubes, reaching the highest productivities above 10 g_5-keto-d-fructose_/L/h during feeding. Moreover, the scale-up of the 5-keto-d-fructose production to a 150 L pressurised fermenter was successfully demonstrated using liquid fructose solutions (745 g/L).

**Conclusion:**

We optimised the 5-keto-d-fructose production process and successfully increased titre, yield and productivity. By using solid fructose, we presented a second feeding strategy, which can be of great interest for further scale-up experiments. A first scale-up of this process was performed, showing the possibility for an industrial production of 5-keto-d-fructose.

**Supplementary Information:**

The online version contains supplementary material available at 10.1186/s12934-022-01980-5.

## Background

Sugars are sweetening compounds that were once seen as the most important achievement for the food industry. In recent decades, more and more diseases have been attributed to excessive sugar consumption like diabetes, obesity, high blood pressure or cardiovascular diseases [1–4]. Therefore, the food industry thrives to discover low-caloric sweeteners. Sweeteners are divided into synthetic and natural sweeteners, including nutritive and non-nutritive sweeteners [1, 5]. The safe consumption of synthetic sweeteners is controversially discussed [5, 6]. Hence, the food industry constantly looks for new, low-caloric natural sweeteners. The global sweeteners market has significantly increased in the last decades and is likely to increase further [7]. It is estimated to reach over $39 billion by 2026 and a compound annual growth rate (CAGR) of 4.5% between 2017 and 2026 [7, 8]. The market is highly fragmented with key industrial players [7]. To develop a competitive new sweetener, reducing the production cost and finally the retail price is essential [7, 8].

A possible new natural sweetener is 5-keto-d-fructose (5KF). 5KF was already described in the 1960s as a sweet substance produced by several *Acetobacter* strains [9, 10]. 5KF occurs naturally and is found, e.g., in grape must and wine [11, 12]. 5KF has a similar sweet taste quality as fructose [13]. Currently, it is unknown, whether 5KF is digested by the human metabolism or only partially [14]. Recent studies suggest a cytotoxicity of 5KF, which needs to be further investigated [15]. 5KF can also potentially be used in the pharmaceutical industry as a precursor for therapeutic substances, particularly as a starting material for synthesising pyrrolidine iminosugars [16]. These can treat viral infections, cancer, diabetes, and genetic disorders as glucosidase inhibitors [17, 18].

The synthesis of 5KF can be carried out enzymatically [19, 20]. However, the required high purity of the enzymes leads to an expensive process [13]. More recent production methods mainly describe the microbiological production, with the help of fructose dehydrogenase (FDH) from *Gluconobacter japonicus*, using *Gluconobacter oxydans* as the production organism [13, 21]. FDH is a membrane-bound enzyme coupled to the electron transport chain of the cytoplasmic membrane [22, 23]. Unlike other enzymes that can perform the oxidation of fructose to 5KF [19, 20, 24], fructose dehydrogenase from *G. japonicus* is highly specific and only converts fructose to 5KF [22].

The biotechnological production of 5KF was established after the entire genetic sequence of the FDH complex of *G. japonicus* NBRC3260 was sequenced for the first time [25]. For this purpose, *fdhSCL* genes were overexpressed in *G. oxydans*. The heterologously expressed enzyme complex catalysed the oxidation of fructose to 5KF. In previous studies it was demonstrated, that plasmid-based overexpression of the *fdhSCL* genes results in significantly higher enzyme activities, compared to the native host *G. japonicus* [21, 25].

*G. oxydans* has only a low growth yield, due to an inefficient respiratory chain and the restricted cytoplasmatic carbon metabolism [26]. The tricarboxylic acid cycle is incomplete, and the Embden–Meyerhof–Parnas pathway is interrupted [26]. The pentose phosphate pathway and the Entner–Doudoroff pathway are the only two functionally complete central metabolic pathways [12, 26]. However, due to its ability to incompletely oxidise sugars and sugar alcohols by membrane-bound dehydrogenases, *G. oxydans* is an important industrial microorganism in biotechnological production processes. For example, *G. oxydans* is used to oxidise d-sorbitol to l-sorbose as one step in the synthesis of vitamin C [27–29]. Other applications of *G. oxydans* are the production of the diabetes drug miglitol, the production of aliphatic acids, aromatic acids and thiocarboxylic acids and the use of enzymes for the detection of sugars, alcohols and polyols [30–32].

Aerobic microorganisms like *G. oxydans*, used in industrial fermentation processes, generally demand a large quantity of oxygen for optimal biomass and product formation [33–36]. The production of 5KF is stoichiometrically coupled with oxygen consumption [13, 21]. Thus, the oxygen demand for 5KF production consists of the oxygen required for the growth of *G. oxydans* and the conversion of fructose to 5KF [13, 37–39].

For an economical production of 5KF high product concentrations are necessary. But high substrate concentrations inhibit the growth rate of *G. oxydans* and product formation, due to osmotic stress [13, 40]. To overcome this issue, a fed-batch process to produce 5KF using *G. oxydans* 621H Δ*hsdR* pBBR1-p264-*fdhSCL*-ST (*G. oxydans fdh*) was developed [13]. More precisely, this process is an extended batch fermentation. In a classical fed-batch process, the substrate is fed at a rate that the substrate concentration is limiting (in the order of the K_m_-value of the substrate). This operating mode is used to avoid substrate inhibition or to allow catabolite repressed product formation. In contrast, in an extended batch process the substrate concentration is not limiting (significantly larger than the K_m_-value). The extended batch cultivation avoids an excessively high initial substrate concentration and maintains a moderate substrate concentration in the fermentation broth. The extended batch fermentation was divided into three parts. Batch cultivation was started with an initial fructose concentration of 150 g/L in a 2 L fermenter [13]. Thereafter, a 1035 g/L fructose solution was fed with a constant feed rate of 26.6 g/h fructose. During the feed phase, cell activity declined, indicated by a stagnant or even decreasing volume-specific respiration [13]. Also, the dissolved oxygen tension (DOT) decreased to 0% during the feed phase. Hence, for a period of 7 h oxygen limitation occurred. After the feed phase, residual fructose was converted to 5KF in a final batch. During this fermentation, a 5KF titre of 489 g/L and a yield of 0.92 g_5KF_/g_fructose_ was achieved [13]. An overall productivity of 7 g_5KF_/L/h was reached [13].

The aim of this work was the optimisation of the 5KF production regarding titre, yield and productivity. Therefore, different process aspects were analysed, including possibilities as substrate limitation, inhibition by substrate or product formation, excessive osmotic pressure and feeding strategy. In addition, a first scale-up was performed.

## Results and discussion

### Investigation of oxygen and second substrate limitation during 5KF production

The oxygen demand for 5KF production is composed of the oxygen required for the aerobic metabolism *G. oxydans fdh* and the conversion of fructose to 5KF [13, 37–39], as production of 5KF is stoichiometrically coupled with oxygen consumption [13, 21]. Oxygen limitation can become a growth-limiting factor, but, the effects on microbial growth and product formation are not clear [28, 39]. During a previously reported fermentation, reaching a titre of 489 g/L, the DOT decreased to 0% for approx. 7 h [13]. Moreover, the oxygen transfer rate (OTR) decreased during the period of oxygen limitation. Usually, the OTR shows a plateau, if oxygen limitation occurs [41]. A decreasing OTR could indicate a second substrate limitation [41]. To make this process more efficient and, therefore, 5KF a competitive new sweetener, it is of great interest to ensure that no limitations occur. Thus, it was investigated, if the decreased respiratory activity was caused by an oxygen or second substrate limitation.

**Fig. 1:**
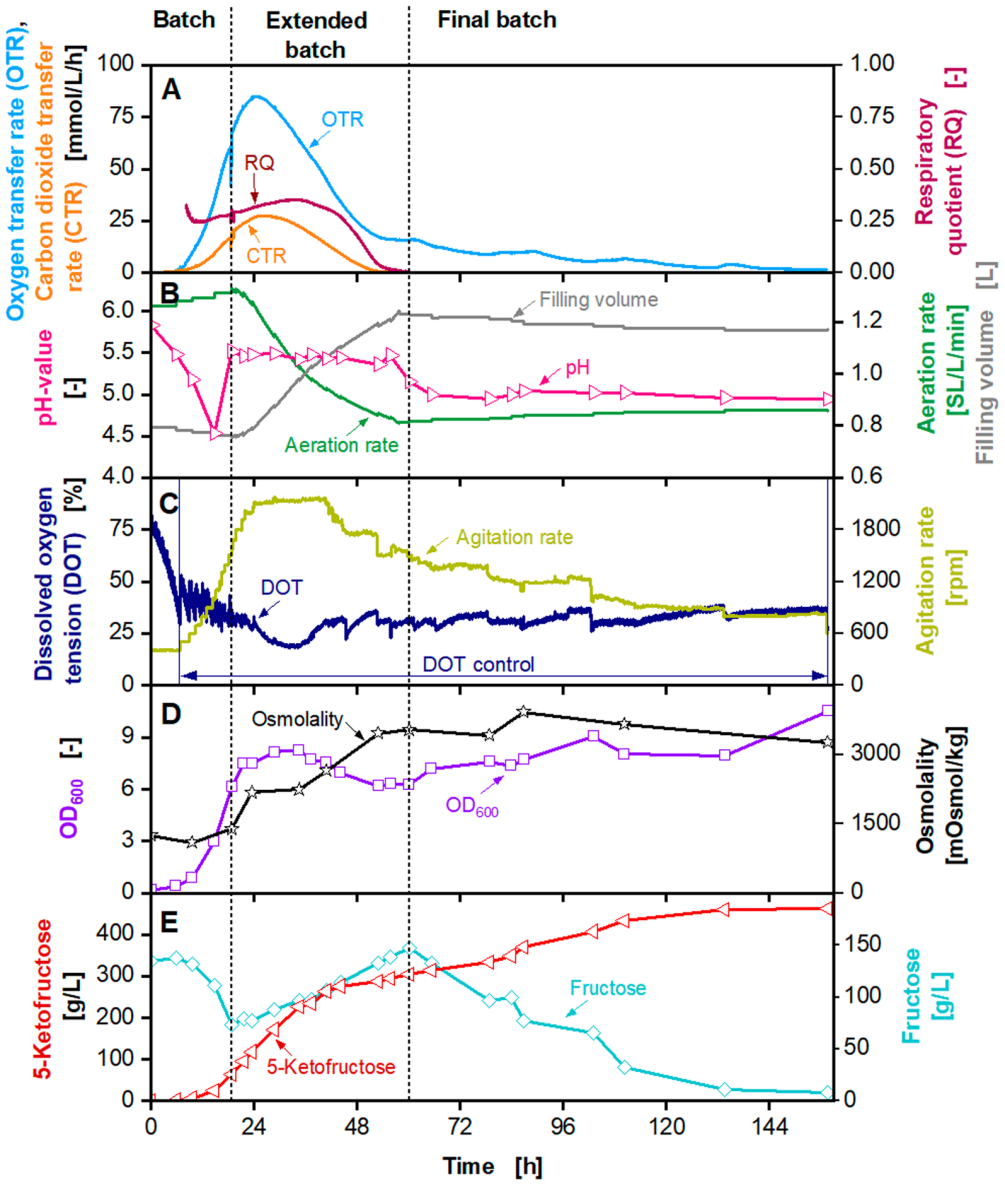
Extended-batch-cultivation of *G. oxydans* 621H Δ*hsdR* pBBR1p264-*fdhSCL*-ST in a 2 L Visual Safety Fermenter (VSF, Bioengineering) with constant feeding of fructose (1035 g/L) between 19 and 61 h. Depicted is **A** the oxygen transfer rate (OTR, light blue), carbon dioxide transfer rate (CTR, orange) and respiratory quotient (RQ, dark red), **B** pH (pink), aeration rate (green) and filling volume (grey), **C** the dissolved oxygen tension (DOT, dark blue) and agitation rate (light green), **D** the optical density OD_600_ (purple) and osmolality (black), **E** fructose (light blue) and 5-ketofructose concentration (red). Cultivation was performed in complex medium with 150 g/L initial fructose at 30 °C, initial pH value 6, pH control at 5.5 from 19–61 h and 5 from 61 h with 3 M KOH, V_L,start_ = 0.8 L in a 2 L fermenter. DOT was kept ≥ 30% by variation of agitation speed (500–2050 rpm), absolute aeration rate Q_g_ = 1 SL/min. Feeding solution: 1035 $\pm$ 10 g_fructose_/L, heat pretreatment: 121 °C, 21 min. Feed rate: 14.3 g_fructose_/h. Fructose feeding solution and peripheral feeding system were heated to ~ 55 °C. RQ-values are only shown, when OTR-values are above 5 mmol/L/h

A 2 L VSF fermenter (Bioengineering) with a maximum agitation rate of more than 2300 rpm was used to overcome the oxygen limitation. The results of the fermentation in the 2 L VSF (Bioengineering) with a feed concentration of 1035 g/L fructose solution are displayed in Fig. 1. The achieved results were similar compared to the previously reported results [13]. During the feed phase, OTR and carbon dioxide transfer rate (CTR) increased to a maximum of 85 mmol/L/h and 28 mmol/L/h, respectively. The optical density measured at 600 nm (OD_600_) increased to 8.3 and the osmolality increased to a maximum above 3500 mOsmol/kg. The DOT was kept above 25% during the fermentation by increasing the agitation rate to 2050 rpm. Due to a technical malfunction of the feed pump, the fructose feed rate was 14 g/h, instead of the intended 26 g/h [13]. Therefore, the feed phase was prolonged from 18 to 60 h. A 5KF titre of 465 g/L was reached during this fermentation, which is in the range of the previously reported titre [13]. No oxygen limitation occurred during this fermentation, in contrast to the previously reported fermentation [13]. Nevertheless, the OTR decreased during the feed phase. Consequently, a short period of oxygen limitation presumably did not influence the 5KF production [13], although the process is highly oxygen-dependent. Hence, a second substrate limitation was investigated.

The medium for 5KF production contains yeast extract, MgSO_4_, (NH_4_)_2_SO_4_ and KH_2_PO_4_. A sufficient amount of yeast extract promotes the growth of *G. oxydans* to higher cell densities [42]. As it was not clear, which component might be limiting, the concentration of the complete medium was increased. First experiments were conducted in shake flasks using the Respiration Activity MOnitoring System (RAMOS) system [41, 43]. The concentration of the medium was increased 1.4-, 1.6-, and 1.8-fold. The initial fructose concentration was kept constant at 150 g/L fructose. The OTR and final OD_600_ of these cultivations are shown in Additional file 1: Fig. S1. It can clearly be seen that the cultivation is oxygen-limited in shake flasks [13]. The maximum oxygen transfer capacity (OTR_max_) in shake flasks can be calculated based on the medium osmolality [44]. The calculated OTR_max_ of 51 mmol/L/h agrees well with the experimental results. The OD_600_ and yield measured at the end of the cultivation showed maximum values for the cultivation using a 1.6-fold increased medium concentration. But the increase in OD_600_ and yield was far less than the 60% increase in medium concentration.

A fermentation was performed in the 2 L VSF (Bioengineering) with a 1.6-fold increased medium concentration (Additional file 1: Fig. S2). No oxygen limitation was detected by increasing the stirrer speed up to 2050 rpm. The maximum OTR increased to 100 mmol/L/h and the maximum OD_600_ to 10.3. Compared with the previously published result by Herweg et al. [13], the 5KF titre with 454 g/L and the yield with 0.87 g_5KF_/g_fructose_ are in the same range. The overall productivity, on the other hand, decreased to 4.1 g_5KF_/L/h and the overall cultivation time increased to 112 h. Especially, the final batch was prolonged for cultivations displayed in Fig. 1 and Additional file 1: Fig. S2. The fructose feed solutions used in those fermentations were autoclaved, which resulted in a brownish colour of the feed solution. This indicates the degradation of the fructose by the Maillard reaction or caramelisation [45–47]. Degradation products could lead to an inhibition of the fermentation process, resulting in decreased productivities. This is discussed in detail in Fig. 8, including all fermentations displayed in this study. The OTR decreased during the extended batch, as described in the previous experiment (Fig. 1). As the medium concentration was increased 1.6 times, a second substrate limitation was excluded as a possibility.

The conducted experiments showed that an oxygen or second substrate limitation could be excluded as reasons for the decreasing respiration. Moreover, a short period of oxygen limitation, as described previously [13], does not affect 5KF production, showing the robustness of this process. Increasing the medium concentration by 1.6-fold increased the biomass formation by 20%, even though it did not increase the productivity in this experiment, as suggested previously [13]. Another possibility for the decreasing respiration is inhibition caused by 5KF or osmolality. During the feed phase, 5KF concentration and osmolality increased simultaneously. In contrast, productivity decreased during the feed phase. As this paper aimed to increase the efficiency of the 5KF production, an inhibition caused by 5KF, or osmolality was investigated in detail, as described in Fig. 5.

### Analysis of the viscosity of fructose solutions with different concentrations at different temperatures

The 5KF production process was designed as an extended batch, using highly concentrated fructose solutions [13]. By applying a feed strategy with a liquid feed solution, not just the substrate fructose is fed into the fermenter, but also water. Hence, the fermentation broth is diluted with water, consequently decreasing the product titre. Using highly concentrated feed solutions, the dilution of the fermentation broth with water is reduced, leading to higher product titres. When increasing the fructose concentration, one key factor is the viscosity, as the feed solutions need to be pumped into the fermenter.

**Fig. 2:**
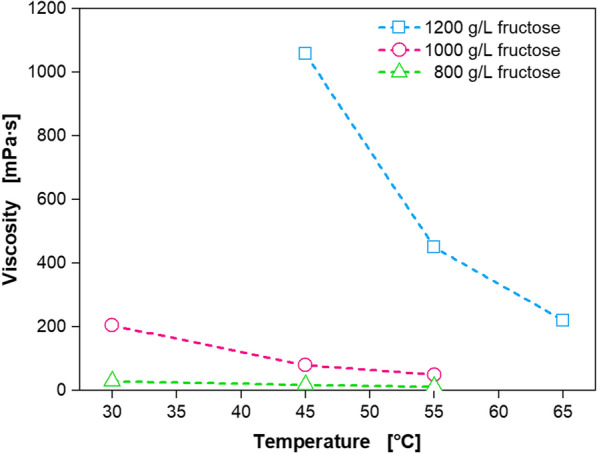
Correlation of viscosity and temperature of fructose solutions with different concentrations. Depicted is the correlation of viscosity and temperature for fructose solutions with concentrations of 800 g_fructose_/L (green), 1000 g_fructose_/L (pink) and 1200 g_fructose_/L (light blue). Fructose solutions were preheated before viscosity measurement. Viscosity was measured using a MCR 301 rheometer (Anton Paar, Stuttgart, Germany) equipped with a cone (CP50-0.5/TG, cone truncation 55 µM, cone angle 0.467°) within the shear rate range of 100–5000 s^−1^. The viscosity can be assumed to be Newtonian within the measured shear range

**Fig. 3:**
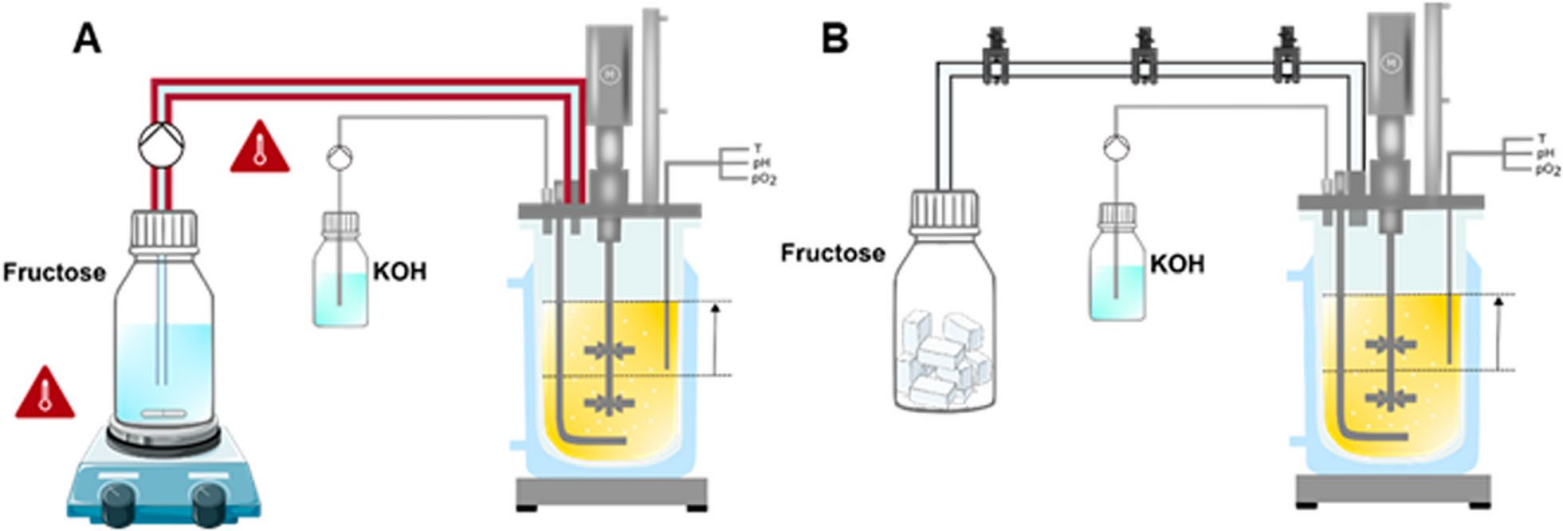
Schematic experimental fermenter setup for concentrated liquid and solid fructose feeding. Extended batch fermentations were performed with **A** concentrated liquid fructose solutions and **B** solid fructose cubes. **A**: Fructose feed solutions were constantly stirred and heated to ~ 55 °C in a bottle using a heating magnetic stirrer. The peripheral feeding system was heated to ~ 55 °C using hot water or an electrical heating band with insulation (indicated with dark red lines). **B**: Fructose cubes were stored in a bottle attached to a silicon tube with a 2 cm diameter. The silicon tube was sealed with three hose clamps, to prevent humidity entering the storage bottle from the fermenter. For feeding, desired numbers of cubes were manually transferred from the bottle to the tube and into the fermenter. Schematic representation adapted from [91]. Parts of the figure were drawn by using pictures form Servier Medical Art, provided by Servier, licensed under a Creative Commons Attribution 3.0 unported license

Figure 2 shows the correlation of viscosity and temperature of fructose solutions with different concentrations. A 800 g/L fructose solution had a viscosity below 30 mPa∙s at 30 °C. In comparison, the viscosity of a 1000 g/L fructose solution is 7 times higher, with 205 mPa∙s at 30 °C. By increasing the temperature to 45 °C and 55 °C, the viscosity was decreased to 80 mPa∙s and 50 mPa∙s, respectively. A fructose feed concentration of 1000 g/L (74% w/w) still contains about a quarter of water, diluting the fermentation broth. Fructose is soluble in water at 20 °C up to 79% w/w [48]. Therefore, attempting to increase the fructose concentration, the fructose solution was heated during preparation to 45 °C. The concentration of the fructose solution was increased by 20% to 1200 g/L (84% w/w). With 1060 mPa∙s the viscosity is 13 times higher than the 1000 g/L solution at the same temperature. Increasing the temperature to 55 °C and 65 °C led to a viscosity of 450 mPa∙s and 220 mPa∙s, respectively. These results agree with previously published viscosity data [48–50]. Thus, by increasing the fructose feed solution from 1000 g/L to 1200 g/L, the amount of water decreased from 26% (w/w) to 16% (w/w). By applying highly concentrated fructose feeding solution, the storage vessel and the peripheral feeding system needed to be heated, to reduce the viscosity of the fructose solution. As illustrated in Fig. 3A, fructose feed solutions were constantly stirred and heated to approx. 55 °C. The peripheral feeding system was heated up to approx. 55 °C using hot water or an electrical heating band with insulation.

### Extended batch fermentation with constant feeding of fructose (1180 g/L)

**Fig. 4:**
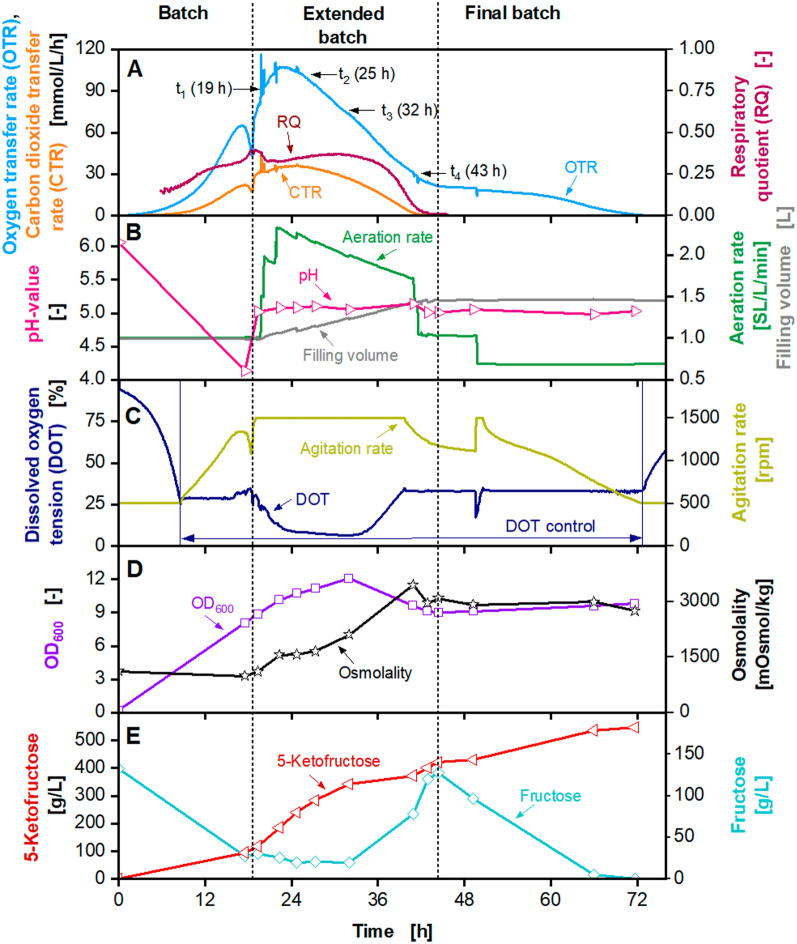
Extended-batch-cultivation of *G. oxydans* 621H Δ*hsdR* pBBR1p264-*fdhSCL*-ST in a 2 L fermenter (Sartorius) with constant feeding of fructose (1180 g/L) between 18 and 44 h. Depicted is **A** the oxygen transfer rate (OTR, light blue), carbon dioxide transfer rate (CTR, orange) and respiratory quotient (RQ, dark red), **B** pH (pink), aeration rate (green) and filling volume (grey), **C** the dissolved oxygen tension (DOT, dark blue) and agitation rate (light green), **D** the optical density OD_600_ (purple) and osmolality (black), **E** fructose (light blue) and 5-ketofructose concentration (red). **A**. Cultivation was performed in complex medium (concentrated 1.6×) with 150 g/L initial fructose at 30 °C, initial pH value 6, pH control at 5 from 18 h with 3 M KOH, V_L,start_ = 1 L in a 2 L fermenter. DOT was kept ≥ 30% by variation of agitation speed (500–1500 rpm), absolute aeration rate Q_g_ = 1—2.5 SL/min. Feeding solution: 1180 $\pm$ 2 g_fructose_/L, heat pretreatment: 100 °C, 10 min. Feed rate: 27.3 g_fructose_/h. Fructose feeding solution and peripheral feeding system were heated to ~ 55 °C. RQ-values are only shown, when OTR-values are above 5 mmol/L/h. Sampling for further experiments Fig. 5) are indicated by time stamps t_1_–t_4_ in **A**. A drift in the oxygen sensor was corrected by linear regression. For original data please refer to Additional file 1: Fig. S3

Overcoming the oxygen limitation, as demonstrated in Fig. 1 and Additional file 1: Fig. S2, didn’t show any improvement of the 5KF titre. However, increasing the medium concentration by 1.6-fold increased biomass formation. Next, it was tested, if the productivity and 5KF titre can be improved by increasing the fructose feed concentration. Hence, a fermentation was conducted with a fructose feed concentration of approx. 1180 g/L. The results are depicted in Fig. 4. A drift in the oxygen sensor was corrected by linear regression. For original data, please refer to Additional file 1: Fig. S3.

**Fig. 5:**
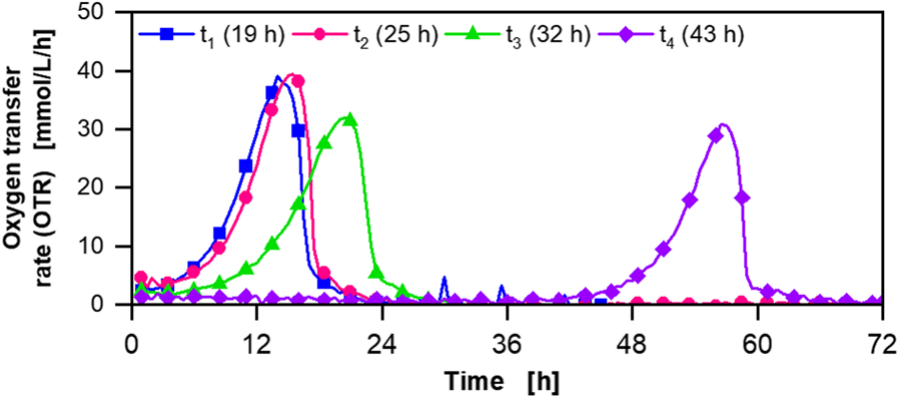
Cultivation of *G. oxydans* 621H Δ*hsdR* pBBR1p264-*fdhSCL*-ST in a RAMOS device with cells sampled at different time points (t_1_–t_4_) from the extended batch fermentation displayed in Fig. 4. Depicted is the oxygen transfer rate (OTR). The samples were taken during the extended batch fermentation (Fig. 4) after 19 h, 25 h, 32 h and 43 h, centrifugated and used for inoculation at an OD_600_ of 0.1. Cultivations were performed at 30 °C, 350 rpm, V_L_ = 10 mL in 250 mL shake flasks, initial pH value 6 and a shaking diameter of 50 mm in complex medium with 80 g/L fructose

The fermentation was started with an initial batch phase with 130 g/L fructose and a pH of 6. During this initial batch phase, OTR and CTR increased to approx. 65 mmol/L/h and 22 mmol/L/h, respectively. The pH decreased to 4.1 and the OD_600_ increased to 8.1. The fructose concentration decreased to 30 g/L after 18 h, and 95 g/L 5KF was produced. The respiratory quotient (RQ) increased to 0.3. After 18 h, the extended batch was started by feeding a 1180 g/L fructose solution. OTR and CTR increased, reaching a maximum of 107 mmol/L/h and 36 mmol/L/h, respectively. Compared to previous fermentations, the pH was maintained at 5 instead of 5.5, to promote the FDH activity [25]. The OD_600_ increased to a maximum of 12.1, followed by a decrease due to the dilution of the fermentation broth. The maximum OTR and CTR are about 20%, and the maximum OD_600_ is about 35% higher than in previous fermentations, indicating increased growth and product formation. The RQ increased slightly from 0.3 to 0.35, then decreased to 0 after 44 h. OTR and CTR decreased until the end of the feed phase to 21 mmol/L/h and 0 mmol/L/h, respectively. The DOT decreased to 30% after 8 h, and the agitation speed increased. At the beginning of the feed phase, the maximum agitation speed of 1500 rpm was reached. Although the aeration rate was manually adjusted to 2.25 SL/L/min, the DOT decreased to a minimum of 6%. After 34 h, the DOT increased to 30%. When anti-foam was added after 49 h, the DOT decreased for a short period.

Due to fructose feeding, the osmolality increased from initially 1100 mOsmol/kg to over 3400 mOsmol/kg. Due to a constant feeding and consumption rate of 27 g/h fructose, the fructose concentration remained at a constant level of approx. 25 g/L. Only at the end of the feed phase, after 40 h, fructose accumulated. 5KF concentration increased during the feed phase. The 5KF productivity decreased after approx. 32 h, as described for the previous fermentations. 5KF concentration increased to 420 g/L and the fructose concentration to 130 g/L after 44 h. During the final batch, the OTR decreased to 0 mmol/L/h, and the residual fructose was converted to 5KF, reaching a final titre of 545 g/L after 72 h. The titre was increased by over 10% compared to the highest 5KF titre reported before [13]. Thus, decreasing the water content of the fructose feed solution successfully increases the 5KF titre. A yield of 0.98 g_5KF_/g_fructose_ and a productivity of 7.6 g_5KF_/L/h was reached. The titre is among the highest reported for *G. oxydans*. E.g. in l-sorbose fermentations, titres up to 628 g/L [28] and in xylonic acid production, titres up to 588 g/L were reached [51, 52]. For xylonic acid fermentation, productivities up to 8.66 g_xylonic acid_/L/h are reported. The 5KF fermentations reached similar results, demonstrating the high efficiency of this production process.

### Characterisation of cell performance in a RAMOS device

To identify the underlying mechanism causing the decreasing respiration activity during the feed phase, the cell performance and viability were analysed using a RAMOS device, as described in the “Materials and methods” section. Cells were sampled from the cultivation in Fig. 4. The OTR curves are depicted in Fig. 5. The first cell sample was taken at the beginning of the feed phase after 19 h (Fig. 4A) when OTR and CTR increased. Shortly after the maximum OTR was reached, the second cell sample was taken after 25 h (Fig. 4A). The cultivation with the cell samples after 19 h and 25 h showed an exponentially increasing OTR with a maximum OTR of 40 mmol/L/h after 14 h and 15.5 h, respectively, followed by a decrease (Fig. 5). Cells sampled after 32 h and 43 h during the feed phase, when OTR and CTR were decreasing (Fig. 4A), showed a maximum OTR of approx. 31 mmol/L/h in RAMOS cultivations (Fig. 5). Moreover, the maximum OTR was reached for the third sample after 20.5 h and for the fourth sample after 57 h.

Cells harvested later during the feed phase (Fig. 4), exhibited a longer lag phase in the RAMOS cultivation. The lag phase was determined, as described before [53], and plotted against the 5KF concentration at the time of sampling (Additional file 1: Fig. S4A). This shows an exponentially increasing relationship between the lag phase and the 5KF concentration. Such a relation was also described during the production of dihydroxyacetone (DHA) [54]. In addition, it could be shown that increasing DHA concentration led to a decrease in cell density and agglomerates. A possible reason for the reduction in cell density is cell lysis. I.e., with increasing DHA concentration, the number of active cells decreased. The results also showed that the inhibitory effect was reversible up to a certain concentration. Product inhibition was also observed in l-erythrulose production, which was indicated by a decrease in the oxygen uptake rate (OUR) with increasing l-erythrulose concentration [55]. For 5KF production, it can be concluded that OTR and CTR decreased during the feed phase due to cell deactivation or cell lysis. Furthermore, the inhibitory effect is reversible for the samples taken during the feed phase. However, it is not clear, whether 5KF is responsible for the inhibition. Another reason for cell deactivation or cell lysis could be an increasing osmolality. An exponentially increasing correlation can be observed, when plotting the lag phase against osmolality (Additional file 1: Fig. S4B). Osmolality is mainly influenced by the concentration of 5KF and fructose in the fermentation broth. The lag phase was plotted against the sum of the concentrations of both substances, which again led to an exponential curve (Additional file 1: Fig. S4C). *G. oxydans* is naturally found in many sugar-rich environments, such as fruit, beer, wine or garden soil [12, 37]. Previous studies showed a high osmotolerance of *G. oxydans* [40, 56]. *G. oxydans* showed growth at glucose concentrations of up to 300 g/L [56] or 200 g/L fructose [13]. During the production of 5KF, osmolality increased to over 3500 mOsmol/kg, which exceeds previous studies of osmotolerance [40, 56]. Mannitol serves as an osmoprotectant in *G. oxydans* [56, 57]. *G. oxydans* produces mannitol intracellularly as a protective agent. Since mannitol can be converted intracellularly in *G. oxydans* by a mannitol dehydrogenase [57], it was assumed that the fructose-containing medium also increased osmotolerance [13].

In summary, reversible inhibition occurred above a concentration of 350 g/L 5KF or above an osmolality of 2100 mOsmol/kg. The decrease in OTR and CTR during the feed phase can be attributed to reduced cell viability. During the fermentation, fructose accumulation can be detected, as seen in previous fermentations (Figs. 1, 4 or Additional file 1: Fig. S2), caused by the same effects. Astonishingly, constant productivities can even be observed after the inhibition of the cells started. In previous research, it was assumed that during the production of DHA, the membrane-bound glycerol-oxidising dehydrogenase was still active in both, the inhibited cells and the cell debris [54]. Further investigations have to be carried out to confirm this hypothesis for 5KF production and the activity of FDH in the cell debris. As 5KF concentration and osmolality increase during the extended batch fermentation, both can cause inhibition. Inhibition could be prevented by in-situ product removal. Moreover, 5KF production could be further optimised by repeated extended batch fermentations, as demonstrated for DHA production [54]. However, it is essential to evaluate, up to which point the cells can be reused.

### Extended batch fermentation with solid fructose feeding

The 5KF titre was increased by 10% by increasing the fructose feed concentration, in comparison to previously publishes results [13]. To feed such highly concentrated fructose solutions, the peripheral feeding system was heated to approx. 55 °C. Due to excessive viscosity, further increase in fructose concentration would be a tremendous technical challenge. Hence, a solid fructose feeding strategy for small scale fermentations was developed. Figure 3B shows the schematic experimental setup for solid fructose feeding. Using fructose cubes instead of fructose powder allowed an easier transfer of fructose into the fermenter. Fructose is hygroscopic and begins to absorb water at low relative humidity [58]. Fructose cubes helped prevent blockages of the silicon tube, due to moisture uptake of fructose. In addition, fructose cubes were easier to dose. The feeding of fructose cubes was conducted by manual DOT controlled fructose pulses.

**Fig. 6:**
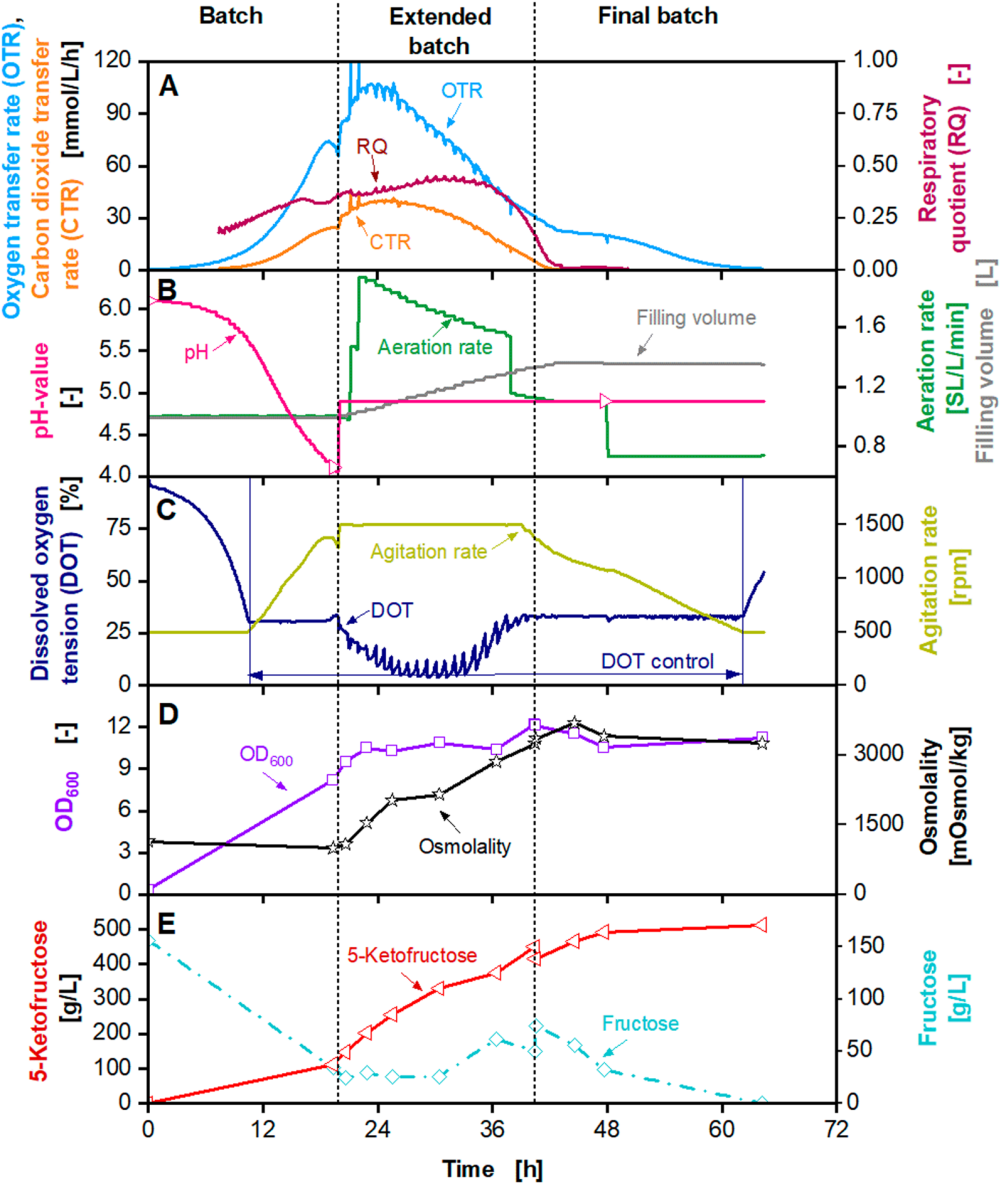
Extended-batch-cultivation of *G. oxydans* 621H Δ*hsdR* pBBR1p264-*fdhSCL*-ST in a 2 L fermenter (Sartorius) with solid fructose feed between 20 and 43 h. Depicted is **A** the oxygen transfer rate (OTR, light blue), carbon dioxide transfer rate (CTR, orange) and respiratory quotient (RQ, dark red), **B** pH (pink), aeration rate (green) and filling volume (grey), **C** the dissolved oxygen tension (DOT, dark blue) and agitation rate (light green), **D** the optical density OD_600_ (purple) and osmolality (black), **E** fructose (light blue) and 5-ketofructose concentration (red). Cultivation was performed in complex medium (concentrated 1.6×) with 150 g/L initial fructose at 30 °C, initial pH value 6, pH control at 5 from 20 h with 10 M KOH, V_L,start_ = 1 L in a 2 L fermenter. DOT was kept ≥ 30% by variation of agitation speed (500–1500 rpm), absolute aeration rate Q_g_ = 1–2.5 SL/min. Feeding: solid fructose cubes. RQ-values are only shown, when OTR-values are above 5 mmol/L/h

A fermentation was conducted with 1.6-fold concentrated main culture medium. The results are depicted in Fig. 6. The fermentation was started with an initial filling volume of 1 L and an initial pH value of 6.0, which was not regulated during the batch phase. The batch phase showed similar results to previous cultivations. The OTR and CTR increased to a local maximum of 75 mmol/L/h and 25 mmol/L/h, respectively. DOT dropped to 30% and was controlled by agitation speed with a maximum of 1500 rpm, and pH dropped to 4. The OD_600_ increased to 8.2. The initial fructose concentration of approx. 150 g/L decreased to 34 g/L, and 111 g/L 5KF were produced. During the feed phase, OTR and CTR increased to a maximum of 103 mmol/L/h and 40 mmol/L/h, respectively, followed by a decrease. The RQ increased from 0.3 to 0.4 during the feed phase, followed by a decline. The initial aeration rate of 1 SL/L/min was manually increased to 2.25 SL/L/min, when DOT dropped below 30%. The OD_600_ increased to a maximum of 12.1. As the dilution was minimised during the feed phase, only a slight decrease in OD_600_ was detected during the feed phase. The osmolality increased to above 3400 mOsmol/kg.

Fructose pulsed feeding was started after 19 h. Thereafter, fructose concentration was only presumed, as samples were not taken before and after every fructose pulse. As the DOT was below 30%, fructose cubes were manually added, as soon as the DOT increased. The increasing DOT indicated a limited availability of fructose. This led to a sawtooth-shaped DOT curve and an average fructose concentration of 25 g/L. Thus, fructose cubes were added 24 times over 23 h, as seen in the sawtooth-shaped DOT and OTR. During the feeding process, an abrasion of the fructose cubes was observed. The cubes lost weight due to the movement of the feeding bottle. In average, 26.9 $\pm$ 2.2 g fructose was manually added to the fermenter at each pulse. This led to an average feed rate of 28 g/h fructose. In total, 677 fructose cubes were added during the fermentation. At the end of the fermentation, after 64 h, a 5KF titre of 511 g/L and a product yield of 0.89 $\pm$ 0.09 g_5KF_/g_fructose_ was reached. The overall productivity was increased to 8 g_5KF_/L/h. It was shown that DOT controlled feeding could be used for 5KF production. Moreover, a simple technique for solid feeding in a small-scale lab fermenter was demonstrated. Preparation of fructose cubes and feeding was labour intense in the small scale. Still, it is a good alternative for scale-up, as the peripheral feeding system and the storage vessel do not need to be heated, and solid fructose feeding can be automated.

### Scale-up of the extended batch fermentation to 50 L and 150 L pressurised fermenter

The next step in 5KF process development was the scale-up. Scale-up is often challenging, as many factors are changing at the same time, e.g. reactor geometry, specific power input (P/V_L_), impeller tip speed or the oxygen mass transfer coefficient (k_L_a) [36]. The estimation of the impact of those parameters on the process performance is of great interest [59]. It is impossible to scale up a bioprocess keeping all process parameters constant [36, 60]. Hence, it is necessary to choose a critical parameter, which has the most crucial influence on the bioprocess [35, 36]. For aerobic processes, aeration and agitation allow an effective mass transfer of oxygen from the gas phase to the liquid medium [35, 36]. For *G. oxydans*, it was reported that mixing and oxygen transfer are the critical parameters for scale-up [39].

A previous study demonstrated that the used 2 L fermenter (Sartorius) could not provide a sufficient oxygen supply for 5KF production, as agitation and aeration rate could not further be increased [13]. Oxygen limitation was overcome by using the 2 L VSF (Bioengineering). The 2 L VSF (Bioengineering) can reach specific power inputs of approx. 40–50 kW/m^3^ [61]. Maximum specific power inputs in the pilot-scale fermenter (approx. 1–5 m^3^) are in the range of up to 3 kW/m^3^ [62]. However, we demonstrated that a short period of oxygen limitation did not influence the production of 5KF. Nevertheless, 5KF production is oxygen dependent, and more extended periods of oxygen limitation will affect the process performance. Many studies demonstrated methods of how to increase the OTR_max_, e.g. using oxygen-enriched air [52, 63], in-situ production of oxygen [64] or oxygen vectors [65–67]. Alternatively, it has been demonstrated that an increase of reactor pressure is also a suitable solution [61, 68, 69]. Protein synthesis is the most sensitive process, which is influenced by pressure [70], but the enzymatic activity can even be enhanced using pressure [71]. However, significant inhibition only occur at pressures significantly greater than 10 bar [72].

The first scale-up experiment was conducted in a 50 L pressurised stirred tank bioreactor (Bioengineering) (Additional file 1: Fig. S5). As the necessary equipment for a solid feed for the pressure fermenter was not available, a liquid fructose feed solution was used again. The initial filling volume of 18 L was chosen, corresponding to half of the working volume of the fermenter of 36 L. The pre-culture was conducted in batch mode in a 2 L fermenter (Sartorius) with 150 g/L fructose. The fermentation broth was transferred to the 50 L pressurised fermenter (Bioengineering), resulting in a starting concentration of 9 g/L 5KF, 170 g/L fructose, an initial OD_600_ of 0.5 and an osmolality of 1500 mOsmol/kg. During the batch phase, OTR and CTR increased to 60 mmol/L/h and 21 mmol/L/h, respectively, after 14 h. OTR and CTR showed similarly shaped curves during the feed phase, as seen in previous cultivations. Maximum OTR and CTR of 88 mmol/L/h and 33 mmol/L/h were reached, respectively. A feed solution of 1035 g/L fructose was used during the feed phase, as not all parts of the peripheral feeding systems could be heated. The feed rate was adjusted to 540 g/h fructose according to the fermentation volume. The OD_600_ reached a maximum of 11.9. At the end of the extended batch, a fructose concentration of 200 g/L had accumulated. A previously described, initial fructose concentration above 150 g/L inhibit growth and 5KF production [13]. However, fructose was consumed constantly during the final batch, and fructose was completely consumed after 98 h. A 5KF titre of 460 g/L was reached.

When the DOT increased above 60%, headspace overpressure was lowered. This led to a DOT in the range of 30% to 90% during the feed phase. During the final batch, DOT increased above 120%. Growth and product formation of *G. oxydans* can be negatively influenced by excessively high oxygen concentrations [39]. As demonstrated before, OTR and CTR decreased during the feed phase, due to cell deactivation or cell lysis (Figs. 4 and 5).

In Additional file 1: Fig. S5, for clarity noisy data in OTR, CTR and RQ were deleted. Additional file 1: Fig. S6 shows the raw data. The green inlay shows RQ and headspace overpressure from 30.15 to 30.25 h, illustrating the origin of the noisy data. The reactor pressure is controlled by a regulator valve in the gas outlet, directly followed by the exhaust gas analyser [68]. When the headspace overpressure is, e.g., reduced from 2 bar to 1.8 bar, this results in a short oscillation of the off-gas values, as shown here for the RQ.

**Fig. 7:**
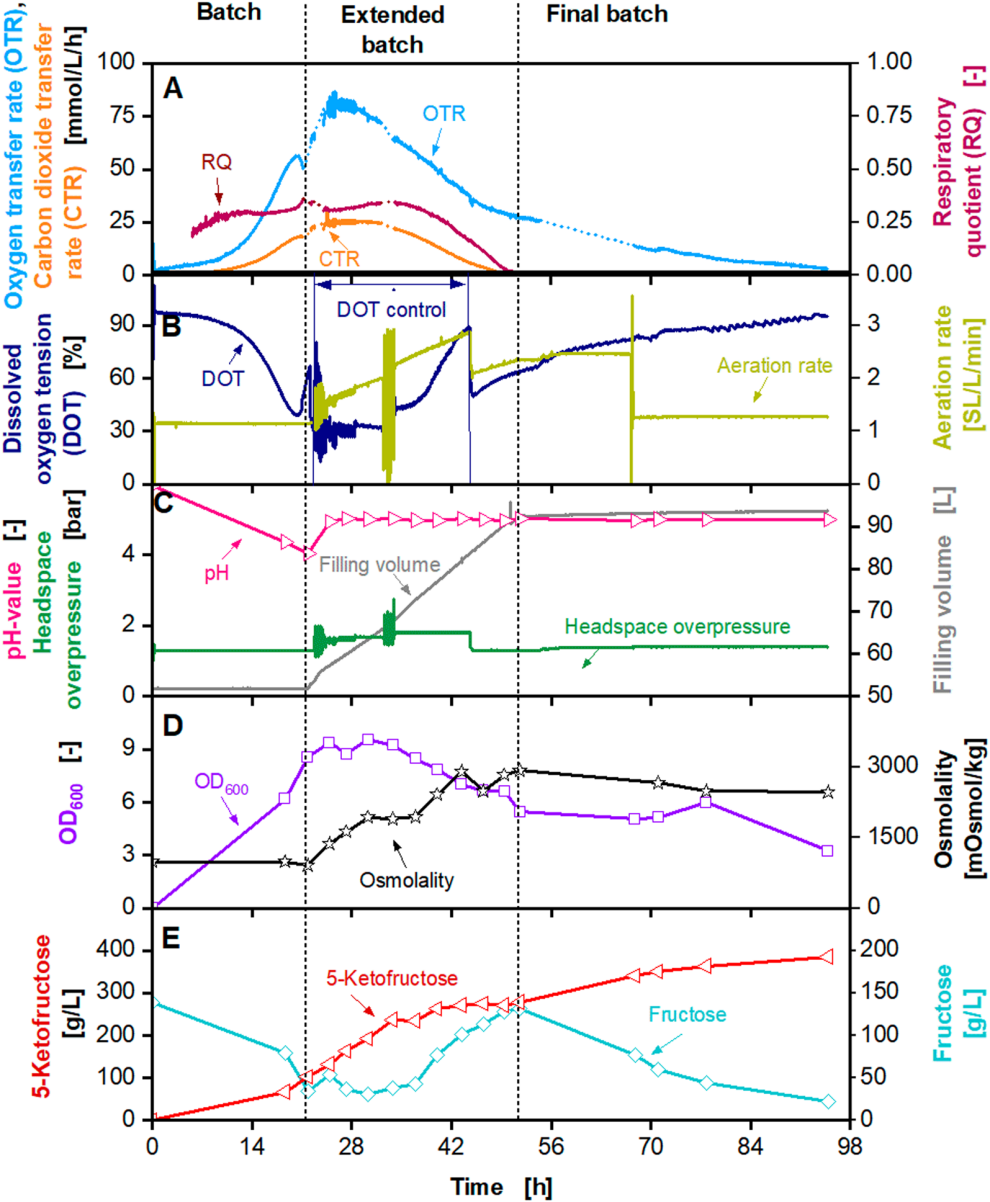
Extended-batch-cultivation of *G. oxydans* 621H Δ*hsdR* pBBR1p264-*fdhSCL*-ST in a 150 L pressurised fermenter (Frings) with constant feeding of fructose (745 g/L) between 22 and 52 h. Depicted is **A** the oxygen transfer rate (OTR, light blue), carbon dioxide transfer rate (CTR, orange) and respiratory quotient (RQ, dark red), **B** the dissolved oxygen tension (DOT, dark blue) and aeration rate (light green), **C** pH (pink), headspace overpressure (green) and filling volume (grey), **D** the optical density OD_600_ (purple) and osmolality (black), **E** fructose (light blue) and 5-ketofructose concentration (red). Cultivation was performed in complex medium (concentrated 1.6×) with 150 g/L initial fructose at 30 °C, initial pH value 6, pH control at 5 from 22 h with 3 M KOH, V_L,start_ = 50 L in a 150 L pressurised fermenter (Frings). DOT was kept ≥ 30% by variation of headspace overpressure (1–1.8 bar), agitation rate: 600 rpm, absolute aeration rate Q_g_ = 50–170 SL/min was increased linearly in parallel to the headspace overpressure. Feeding solution: 745 $\pm$ 25 g_fructose_/L, heat pretreatment: 100 °C, 4 h. Feed rate: 900 g_fructose_/h. Fructose feeding solution was heated to ~ 50 °C. RQ-values are only shown, when OTR-values are above 5 mmol/L/h. Due to technical problems between 22 and 26 h, 32 and 35 h, and 54 and 66 h, OTR, CTR and RQ data shown in dashed lines are not reliable and were approximated by straight lines. For raw data please refer to Additional file 1: Fig. S7

The maximum titre of 460 g/L 5KF is in the range of fermentations performed at 2 L scale with similar concentrated feed solutions (Fig. 1, Additional file 1: Fig. S2) [13]. Hence, a first successful scale-up was demonstrated. During the fermentation, the power consumption of the stirrer was determined by measuring the torque. The specific power consumption was calculated as described previously [61] and was in a range of 1–5 kW/m^3^. The next scale-up step was performed into a 150 L pressurised fermenter (Frings) with a working volume of 100 L (Fig. 7). The initial filling volume was set to 50 L. The cultivation was started with an initial fructose concentration of 140 g/L, an initial OD_600_ of 0.1 and an osmolality of 980 mOsmol/kg. The agitation rate was set to 600 rpm, which resulted in a maximum specific power input of approx. 5 kW/m^3^ for maximum filling volume. DOT was controlled at 30% by the headspace overpressure. The initial volumetric aeration rate was set to 1 SL/L/min and was increased linearly to the headspace overpressure. Due to technical problems (between 22 and 26 h, 32 and 35 h and 54 and 66 h), OTR, CTR and RQ data shown in dashed lines are not reliable and were approximated by straight lines. For raw data and an explanation of technical problems, please refer to Additional file 1: Fig. S7. OTR and CTR increased exponentially during the batch phase and reached a local maximum of 55 mmol/L/h and 18 mmol/L/h after 20 h, respectively (Fig. 7). The batch phase was slightly prolonged, compared to the cultivations in the 2 L fermenters. At the end of the batch phase, after 22 h, fructose concentration decreased to 24 g/L, and 102 g/L 5KF was produced. During the fermentation, no heating of the peripheral feeding system was possible. Therefore, during the feed phase a fructose feed concentration of approx. 745 g/L and a feed rate of 900 g/h fructose were applied. OTR and CTR reached a maximum of 80 mmol/L/h and 18 mmol/L/h after 25 h, respectively. OTR and CTR decreased, and CTR reached 0 mmol/L/h after 52 h, marking the end of the feeding. The RQ is at approx. 0.3 until it decreased as the CTR decreased. The headspace overpressure was increased to 1.8 bar, maintaining a DOT of 30%. The OD_600_ reached a maximum of 9.5, and the osmolality increased to 3000 mOsmol/kg during the feed phase. The fructose concentration during the first part of the feed phase was maintained between 35 g/L and 40 g/L. After 37 h, the fructose concentration increased to 132 g/L at the end of the extended batch. The accumulation of fructose during the end of the feed phase and the decreasing OTR and CTR were caused by cell deactivation or cell lysis, as explained before. The residual fructose was almost entierly consumed during the final batch, and 385 g/L 5KF were produced. A yield of approx. 0.98 g_5KF_/g_fructose_ was reached and the overall productivity was 4.1 g_5FK_/L/h. In a previously conducted fermentation in a 2 L fermenter (Sartorius) with a fructose feed concentration of 760 g/L a 5KF titre of 411 g/L was reached [13]. Therefore, it can be concluded that the scale-up of the 5KF production into 100 L scale was successfully demonstrated.

### Comparison of productivities, oxygen transfer rates and fructose concentrations for extended batch fermentations of this work using different fructose feed concentrations at different scales

**Fig. 8:**
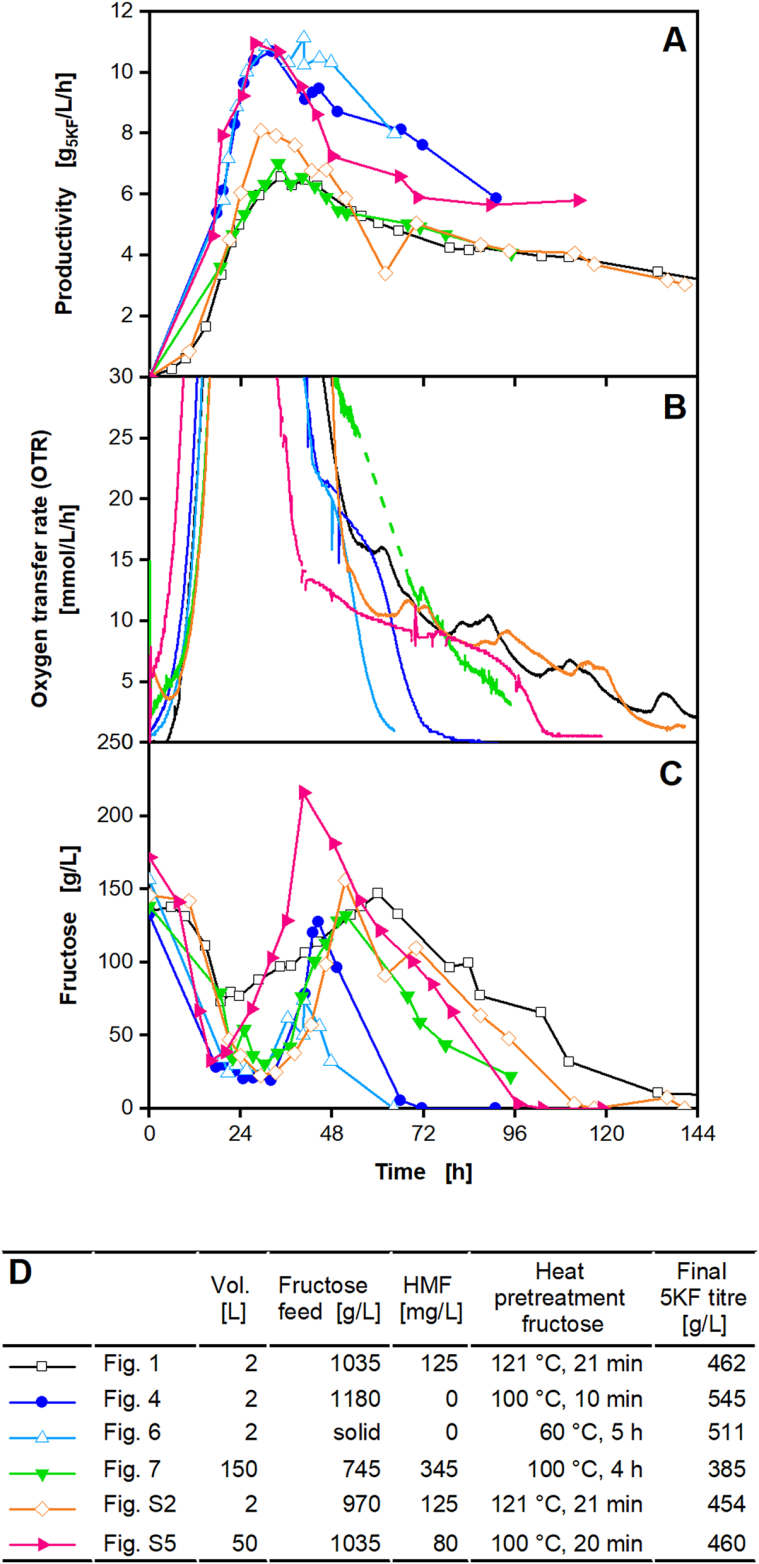
Comparison of productivities, oxygen transfer rates and fructose concentrations for extended batch fermentations of *G. oxydans* 621H Δ*hsdR* pBBR1p264-*fdhSCL*-ST of this work, using different fermenters and fructose feed concentrations. Depicted are **A** productivities **B** oxygen transfer rates, **C** the fructose concentrations and **D** main information for cultivations displayed in Fig. 1 (black), Fig. 4 (blue), Fig. 6 (light blue), Fig. 7 (green), Additional file 1: Fig. S2 (orange) and Additional file 1: Fig. S5 (pink). For better visualisation of OTR curves during final batch phases, y-axis of **B** is only shown for OTR values between 0 and 30 mmol/L/h

All cultivations, performed in this study, were compared, regarding productivity. An overview of all fermentations can be found in Additional file 1: Tab. S1. Figure 8A shows the productivity over time (Eq. 4). For all fermentations, productivity increased until approx. 27 h to 33 h and decreased afterwards. The decrease occurred around the same time due to inhibition at elevated 5KF concentration or osmolality. The fermentation in a 50 L pressurised fermenter (Bioengineering) reached the maximum productivity earliest (Fig. 8A). The reason might be the increased OD_600_ of 0.5 at the beginning of the fermentation, which was 5 times higher than in the other fermentations. Looking at the increase of the productivity, two trends are visible. Fermentations with onefold concentrated medium and 1035 g/L fructose solution (Fig. 1), with 1.6-fold concentrated medium and 970 g/L fructose solution (Additional file 1: Fig. S2) and in the 150 L fermenter (Fig. 7) showed lower productivities (6.6 and 8.1 g_5KF_/L/h) in comparison with the other cultivations. Looking at the OTR of the final batch phase (Fig. 8B), a prolonged respiration (longer than 96 h) is visible for those three cultivations (Figs. 1, 7 and Additional file 1: Fig. S2) and for the cultivation in the 50 L pressurised fermenter (Bioengineering) (Additional file 1: Fig. S5). This trend can also be recognised by looking at the fructose concentrations over time during the final batch phase (Fig. 8C). The time of the final batch phase was considerably lower in fermentations with higher biomass yield (1.6-fold concentrated medium) and increased fructose supply (1180 g/L fructose solution or solid fructose feeding) (Figs. 4 and 6). Comparing all cultivations, no apparent factor seems to be connecting the cultivations showing a decreased productivity or increased cultivation time. When looking at the preparation methods of the fructose solutions, differences become visible (Fig. 8D). Fructose solutions were autoclaved for the fermentation with a onefold concentrated medium and 1035 g/L fructose solution (Fig. 1) and with a 1.6-fold concentrated medium and 970 g/L fructose solution (Additional file 1: Fig. S2). The fructose solutions were brownish coloured after autoclaving. Due to that, fructose solutions were no longer autoclaved and heated to a maximum temperature of 100 °C. The fructose solution for the 150 L fermentation was heated to 100 °C for 4 h, compared to 10 to 20 min for the 2 L and 50 L scale fermentations. Solid fructose cubes were dried at only 60 °C for 5 h. Thermal treatment of the fructose solution and fructose cubes was used for preparation, not for sterilisation. However, the high osmolality of the fructose solution itself and the osmolalities during fermentation prevented contamination. High osmotic pressure and low water activity are growth limiting factors for many microorganisms [73–76]. E.g., increased osmotic pressure is used to preserve food products [77].

The colouration of heated fructose resulted from the decomposition of hexoses during the heat sterilisation [45, 78–81]. The two pathways involved in thermal degradation are the Maillard reaction and caramelisation [45–47]. A decomposition intermediate of hexoses is 5-hydroxymethylfurfural (HMF) [78], mainly at pH values below 7 [82]. HMF is also a by-product of lignocellulosic pre-treatment and a well-known inhibitor of microorganisms [51, 52, 83–86]. Using biomass hydrolysates as substrates for the cultivation of *G. oxydans* showed inhibitory effects, e.g. during the production of xylonic acid [51, 52]. Therefore, HMF concentrations were measured at the beginning of the fermentations and at different times. Results are displayed in Fig. 8D. Small amounts of HMF were only found in fermentation with a onefold concentrated medium and 1035 g/L fructose solution (Fig. 1), with a 1.6-fold concentrated medium and 970 g/L fructose solution (Additional file 1: Fig. S2) and in the 50 L and the 150 L fermenter (Additional file 1: Fig. S5 and Fig. 7). These fermentations showed a decreased productivity at the beginning of the fermentation, except for the cultivation in the 50 L pressurised fermenter (Bioengineering). As described before, an increased initial OD_600_ was applied during this cultivation. The amounts of HMF were relatively low, but were used as an indicator of fructose degradation. HMF concentration increase with increasing temperature and time of thermal treatment [45]. As some fructose feed solutions were autoclaved at 121 °C, and others were only heated to 100 °C for 10 min, decomposition of fructose is a possible explanation for the inhibition causing decreased productivities. As fructose feed solutions were added during the extended batch, HMF was constantly added. In addition to the inhibition due to 5KF concentration or osmolality occurring during an extended batch, HMF might also inhibit product formation. This might also have elongated the final batch for the fermentation with a onefold concentrated medium and 1035 g/L fructose solution (Fig. 1), with a 1.6-fold concentrated medium and 970 g/L fructose solution (Additional file 1: Fig. S2) and in the 50 L and the 150 L fermenter (Additional file 1: Fig. S5 and Fig. 7).

*G. oxydans* DSM 50049 was able to oxidise HMF to 5-hydroxymethyl-2-furan carboxylic acid (HMFCA), as previously demonstrated [87]. The involvement of a membrane-bound enzyme was suggested. The cultivations were performed with resting cells, and it was concluded that higher cell densities have a protective effect against the inhibitory effects of HMFCA [87]. Until now, it is not clear, which *G. oxydans* strains are able to oxidise HMF [87]. Therefore, it might be interesting to investigate, whether *G. oxydans fdh* is capable of oxidising HMF or not.

The highest productivities were reached for the fermentation with a 1.6-fold concentrated medium and 1180 g/L fructose solution and solid fructose feeding (Figs. 4 and 6) and the 50 L pressurised fermenter (Bioengineering) (Additional file 1: Fig. S5). The productivities increased to 10.8 g_5KF_/L/h after approx. 28 h. Increasing the feed fructose solution concentration resulted in a decreasing dilution, which is also reflected by the increased productivity. The increased initial OD_600_ might also positively influence the productivity during the fermentation in the 50 L pressurised fermenter (Bioengineering). Using solid fructose cubes for feeding resulted in the smallest dilution effect. The productivity showed the smallest decrease as the volume increase during feeding is small and remained above 10 g_5KF_/L/h for more than 30% of the cultivation time. The overall productivity (Eq. 5) was highest for solid fructose feeding with 8 g_5KF_/L/h.

The 5KF production process is very efficient, even though inhibitory effects influence the production of 5KF. A rough cost estimation using the SuperPro Designer software (data not shown) indicates that the production cost for 10,000 t of 5KF per year at a fructose substrate cost of 1.16 $ per kg is in the order of 4.3 $ per kg. Considering the two most potent feeding strategies, using a heated feed solution with approx. 1200 g/L fructose or solid fructose cubes, solid fructose feeding has the advantage that no heating of the peripheral feeding system is necessary.

## Conclusion

In this study, the highly efficient production of the potential new sweetener 5KF, using *G. oxydans fdh*, was analysed and optimised. We could demonstrate that the decreasing respiration during feeding occurred due to inhibition by elevated 5KF concentrations or osmolality. Oxygen or second substrate limitation were excluded as reasons. The inhibitory effect is reversible, but was significantly influenced by 5KF concentrations above 350 g/L or osmolalities above 2100 mOsmol/kg. This information is of great interest for further process optimisation. This could include an in-situ product removal or repeated extended batch fermentations. By increasing the concentration of the fructose feeding solution to approx. 1200 g/L, the 5KF titre was increased by 10% to 545 g/L. This is an outstanding achievement, as this titre is among the highest reported titres in literature. The viscosity of the feeding solutions is of great importance. To feed such a highly concentrated feeding solutions, the solution itself and the peripheral feeding system needed to be heated to approx. 55 °C. Moreover, thermal treatment during fructose solution preparation had to be carefully conducted, as increased temperatures led to fructose degradation and HMF formation. Fructose solutions were not sterilised by autoclaving. Increased osmolalities during fermentation prevented contamination. An alternative feeding strategy was presented using solid fructose cubes. This feeding strategy overcame the problems occurring with liquid feeding solutions and can be of great interest for further scale-up experiments. Moreover, the scale-up to an 150 L fermenter of the 5KF production was successfully demonstrated using liquid fructose solutions.

## Materials and methods

### Microbial strain

The strain *G. oxydans* 621H Δ*hsdR* containing the plasmid pBBR1-p264-*fdhSCL*-ST (*G. oxydans fdh*) was used [21]. The plasmid contained the gene for the membrane-bound enzyme fructose dehydrogenase (FDH, EC 1.1.99.11) and a gene for kanamycin resistance [21, 22, 88]. The strain has a natural resistance to the antibiotic cefoxitin. For strain maintenance, stocks containing 200 g/L glycerol were used and stored at − 80 °C.

### Media composition

In this study, the complex medium used for the main cultivation of *G. oxydans* contained 5 g/L yeast extract (Karl Roth GmbH, Karlsruhe, Germany), 2.5 g/L MgSO_4_·7 H_2_O, 1 g/L (NH_4_)_2_SO_4_ and 1 g/L KH_2_PO_4_ [89]. The initial pH was adjusted to 6 with KOH (3 M or 10 M). The medium was supplemented with 50 µg/mL cefoxitin for pre-cultivations and 50 µg/mL kanamycin for pre-cultivations and main cultivations. Pre-cultures were supplemented with 80 g/L mannitol and main cultures with 150 g/L fructose as substrate, unless otherwise stated. Fructose feed solutions were prepared in concentrations from 745 g/L to 1200 g/L. Exact concentrations are given for each experiment. Fructose feed solutions were prepared with deionised water and either autoclaved or heated to 100 °C.

For solid fructose cube preparation, a mixture of fructose and deionised water was prepared containing 98% (w/w) fructose. The mixture was transferred in a silicone cube form (cube size 9 mm) and dried at 60 °C for approx. 5 h. A control sample of 20 fructose cubes was weighed to estimate the standard deviation before and after the fermentation. Fructose cubes had an edge length of 9 mm and a weight of 0.96 $\pm$ 0.07 g. The fructose cubes were stored in a bottle attached to the fermenter with a silicon tube (inner diameter 1.5 cm) (Fig. 3B). The silicon tube was sealed with three hose clamps to prevent humidity from entering the storage bottle from the fermenter. The desired number of cubes was manually transferred from the bottle to the tube and into the fermenter, for feeding.

### Cultivation in the respiration activity monitoring system (RAMOS)

Shake flasks cultivations were performed at 30 °C in 250 mL shake flasks (unbaffled) with an initial filling volume of 10 mL, at 350 rpm shaking frequency and 50 mm shaking diameter (Climo-Shaker ISF1-X, Kuhner, Birsfelden, Switzerland). The aeration rate was 10 mL/min (1 vvm). Online monitoring of the respiratory activity was performed using the Respiration Activity MOnitoring System (RAMOS) developed at our chair [41, 43]. Commercial versions of the RAMOS device can be acquired from Kühner AG (Birsfeld, Switzerland) or HiTec Zang GmbH (Herzogenrath, Germany). Pre-cultures were inoculated with 100 µL glycerol stock cell suspension (optical density measured at 600 nm (OD_600_) = 2.4) per 10 mL pre-culture medium and cultivated for approx. 16 h. Pre-cultures were centrifuged in sterile 2 mL tubes for 3 min and 14,000 rpm at room temperature and then resuspended in the main culture medium. Main cultures were inoculated with an OD_600_ of 0.1. All RAMOS cultivations were performed in duplicates. Cultivations with *G. oxydans fdh* are highly reproducible. To demonstrate this, we have plotted in Additional file 1: Fig. S8 cultivations with *G. oxydans fdh* with 150 g/L and 1.0× concentrated medium, carried out over a period of more than 4 years. Differences in the lag phases have been corrected by shifting the x-axis by a maximum of 5 h. It can be seen in Additional file 1: Fig. S8 that all OTR curves show the same pattern. There are only differences in the OTR_max_. According to Meier et al. [45], the OTR_max_ depends on the osmolality. The osmolality varies slightly in each experiment, due to small deviations in the preparation of the media and pipetting errors.

### Cultivation in 2 L, 50 L and 150 L bioreactors

Fermentation experiments were performed in a 2 L Visual Safety Fermenter (VSF, Bioengineering AG, Wald, Switzerland), a 2 L Sartorius BIOSTAT^®^ stirred tank reactor (Sartorius, Goettingen, Germany), a 50 L pressurised fermenter (LP 351, Bioengineering AG, Switzerland) and a 150 L pressurised fermenter (Frings Proreact P-Atex, Heinrich Frings GmbH & Co. KG, Rheinbach, Germany) at 30 °C.

Fermentation experiments in the 2 L VSF (Bioengineering) were performed with an initial filling volume of 0.8 L and an aeration rate of 1 SL/min. The VSF was equipped with three six-bladed Rushton turbines (4.8 cm diameter and 1.2 cm height). The baffles consist of two metal coils acting as heat transfer areas for cooling purposes. The DOT (Ingold AP, Mettler-Toledo GmbH, Frankfurt, Germany) was maintained at > 30% by adjusting the agitation rate between 500 and 2200 rpm. The pH value was measured using a pH electrode (EasyFerm Plus K8 200, Hamilton, Hoechst, Germany). The exhaust gas composition was monitored using an Advance Optima Uras 14 exhaust gas analyser (ABB Ltd, Zurich, Switzerland). A peristaltic pump (Reglo analog MS-2/8C, Ismatec, Wertheim, Germany) was used for the fructose feed.

Fermentation experiments in the 2 L fermenter (Sartorius) were performed with an initial filling volume of 1 L and an aeration rate of 1 SL/min. The 2 L fermenter (Sartorius) was equipped with 4 baffles and two 6 bladed Rushton turbines (5.8 cm diameter and 1.1 cm height). The DOT (VisiFermTM DO 225 pO_2_ sensor, Hamilton, Hoechst, Germany) was maintained at > 30% by adjusting the agitation rate between 500 and 1500 rpm. The pH value was measured using a pH electrode (EasyFerm Plus K8 200, Hamilton, Hoechst, Germany). The exhaust gas composition was monitored using a DASGIP G4 exhaust gas analyser (Eppendorf, Wesseling-Berzdorf, Germany). A peristaltic pump (101 U/R, Watson-Marlow Pump Group, Falmouth, UK) was used for the fructose feed.

To analyse the cell performance and viability, fermentation broth samples were taken during the feed phase at four times. Cells were centrifuged and used to inoculate fresh complex medium with 80 g/L fructose. Cultivations were performed in shake flasks using the RAMOS system, as described above.

The fermentation experiment in a 50 L pressurised fermenter was performed with an initial filling volume of 18 L and an initial aeration rate of 18 SL/min. The headspace pressure can be increased up to 10 bar (overpressure). The fermenter was equipped with 4 baffles and three 6 bladed Rushton turbines (12 cm diameter and 2.5 cm height) [68]. The DOT (InPro 6800, Mettler-Toledo GmbH, Gießen, Germany) was maintained at > 30% by adjusting the headspace overpressure between 0.2 and 4 bar. The gas flow rate (standard condition) was increased linearly to the headspace pressure, to keep a constant superficial gas velocity of 18 SL/min inside the reactor [68, 90]. The k_L_a value in a stirred tank reactor remains constant, regardless of the reactor pressure, if the superficial gas velocity in the reactor is kept constant [68]. The agitation rate was set to 500 rpm. The pH value was measured using a pH electrode (405-DPAS-SC-K8C, Mettler-Toledo GmbH, Frankfurt, Germany). The exhaust gas composition was monitored using a Rosemount NGA 2000 exhaust gas analyser (Emerson Automation Solutions, Langenfeld, Germany). Diaphragm pumps (CMS 1804V EXT, Bioengineering AG, Wald, Switzerland and CMS Digital 1804, Dosatronic GmbH, Ravensburg, Germany) were connected to the fermenter and used for pH adjustment and fructose feed, respectively.

Fermentation experiments in a 150 L pressurised fermenter were performed with an initial filling volume of 50 L and an initial aeration rate of 50 SL/min. The headspace pressure can be increased up to 10 bar (overpressure). The fermenter was equipped with 4 baffles and three 6 bladed Rushton turbines (15.8 cm diameter and 3 cm height). The DOT (VisiPro DO Ex 120 H2, Hamilton, Hoechst, Germany) was maintained at > 30% by adjusting the headspace overpressure between 0.2 and 1 bar. The gas flow rate was increased linearly to the headspace pressure to maintain a constant superficial gas velocity inside the reactor of 50 SL/min [68, 90]. The agitation rate was set to 600 rpm. The pH value was measured using a pH electrode (Polilyte Plus H VP 120 Pt100, Hamilton, Hoechst, Germany). The exhaust gas composition was monitored using a Rosemount™ X-STREAM XEFD exhaust gas analyser (Emerson Automation Solutions, Langenfeld, Germany). Diaphragm pumps (LEC-M316S and LEB-M316S, Lewa GmbH, Leonberg, Germany) were connected to the fermenter and used for pH adjustment and fructose feed.

Pre-cultures for 50 L and 150 L pressurised fermenters were conducted in the 2 L fermenter (Sartorius) using the setup described before. Batch cultivations with 1 L initial filling volume were performed with 150 g/L fructose as substrate. Pre-culture was transferred to the main fermenter. The resulting volume change was taken into account regarding the initial filling volume and media composition.

After the initial batch phase, the pH was controlled at 5 with a 3 M KOH solution during all fermentations, unless otherwise stated. To prevent foaming, 0.5 mL antifoam agent Plurafac LF 1300 (BASF, Ludwigshafen, Germany) was added at the beginning of each experiment and when needed. During fermentation, samples were taken from the bioreactor for offline analysis. Volume change by KOH titration and sampling were considered for mass balancing.

### Offline analyses

Samples taken during experiments were analysed regarding pH, OD_600_, osmolality, fructose and 5KF concentrations. The pH was measured with a HI221 Basic pH (Hanna Instruments Deutschland GmbH, Vöhringen, Germany), calibrated with two standard buffer solutions at pH 4 and 7. OD_600_ was measured using a Genesys 20 photometer (Thermo Scientific, Darmstadt, Germany) with a linear range between 0.1 and 0.3. Samples were diluted with 0.9% (w/v) NaCl, if necessary. The osmolality was determined using the cryoscopic Osmometer OSMOMAT^®^ 030 (Genotec, Berlin, Germany). Fructose and 5KF concentrations were analysed by high-performance liquid chromatography (HPLC) measurement (Prominence HPLC system, Shimadzu, Duisburg, Germany or Dionex UltiMate 3000, Thermo Scientific, Darmstadt, Germany). The HPLC systems were equipped with the following columns and detectors: precolumn Organic Acid Resin (40 × 8 mm, CS-Chromatography, Service, Langerwehe, Germany), column Organic Acid Resin (250 × 8 mm, CS-Chromatography Services, Langerwehe, Germany), detector RID-20A Refraktometer (Shimadzu, Duisburg, Germany). The mobile phase consisted of 5 mM H_2_SO_4_. The flow rate was adjusted to 0.8 mL/min at 30 °C and an injection volume of 20 µL. Prior to analysis, samples were centrifuged at 17,000*g* for 4 min. The supernatant was diluted with deionised water, filtered (0.2 µm syringe filter, Whatman™, GE Healthcare, Freiburg, Germany) and heated to 60 °C for 60 min (avoiding a double peak, probably caused by the existence of 5KF in an equilibrium of the keto and the germinal diol form) [13]. 5-Hydroxymethylfurfural (HMF) concentrations were measured by HPLC (Prominence HPLC system, Shimadzu, Duisburg, Germany). The HPLC system was equipped with the following columns and detectors: precolumn Organic Acid Resin (40 × 8 mm, CS-Chromatography, Service, Langerwehe, Germany), column Organic Acid Resin (250 × 8 mm, CS-Chromatography Services, Langerwehe, Germany), detector RID-20A Refractometer (Shimadzu, Duisburg, Germany). The mobile phase consisted of 5 mM H_2_SO_4_. The flow rate was adjusted to 0.8 mL/min at 40 °C and an injection volume of 20 µL. Viscosity was measured using a MCR 301 rheometer (Anton Paar, Stuttgart, Germany) equipped with a cone (CP50-0.5/TG, cone truncation 54 µM, cone angle 0.467°) within the shear rate range of 100–5000 1/s at different temperatures between 30 and 65 °C. Fructose solutions with concentrations between 800 and 1200 g/L were prepared as described before and preheated to the measuring temperature between 30 and 65 °C.

### Calculations

Specific parameters were calculated and compared for the experiments. This includes the yield Y in g_5KF_/g_fructose_ and the productivity. Samples taken during cultivations as well as the addition of base for titration or anti-foam agents were considered and included in the mass balancing.

The overall yield Y [g_5KF_/g_fructose_] was calculated by dividing the mass of the produced 5KF by the mass of fructose (Eq. 1).

1 $$Y ({t}_{end}) = \frac{{m}_{5KF}({t}_{end})}{{m}_{F}({t}_{end})}$$

For the calculation of the yield Y [g_5KF_/g_fructose_], the mass of fructose m_F_(t) [g] (Eq. 2) and 5KF m_5FK_(t) [g] (Eq. 3) needed to be calculated.

2 $${m}_{F} \left(t\right)= {c}_{F} \left({t}_{0}\right)\cdot {V}_{L} ({t}_{0})+ {c}_{feed}\cdot {V}_{feed} \left(t\right)- \sum_{{t}_{0}}^{t}{c}_{F}\left(t\right)\cdot {V}_{sample}(t)$$

Equation 2 considers the initial fructose concentration c_F_(t_0_) [g/L] and the initial filling volume V_L_(t_0_) [L]. The addition of fructose during feeding is considered as the feed concentration c_feed_ and the added feed amount V_feed_(t) [L] at a specific time point (t). Sampling during the fermentation was considered, using the sum of the fructose concentration c_F_(t) [g/L] times the volume of the taken sample V_sample_(t) [L].

3 $${m}_{5KF} \left(t\right)= {c}_{5kF} \left(t\right)\cdot {V}_{L}\left(t\right)+ \sum_{{t}_{0}}^{t}{c}_{5KF}\left(t\right)\cdot {V}_{sample}(t)$$

Equation 3 calculates m_5KF_ from the 5KF concentration c_5KF_(t_0_) [g/L] and the filling Volume V_L_(t) [L] at a specific time point (t). Sampling during the fermentation was taken into account, using the sum of the 5KF concentration c_5KF_(t) [g/L] times the volume of the taken sample V_sample_(t) [L].

The productivity (Eq. 4) was calculated at different time points (t) during the fermentation and at the end of the fermentation (overall productivity, Eq. 5). The total mass of 5KF at a specific time point or at the end of the fermentation was divided by the sum of the filling volume V_L_(t) and the sample volume V_sample_(t) times the fermentation time t [h].

4 $$Productivity (t)= \frac{{m}_{5KF}(t) }{\left({ V}_{L}\left(t\right)+ {V}_{sample}\left(t\right)\right)\cdot t}$$

5 $$Overall Productivity ({t}_{end})= \frac{{m}_{5KF}({t}_{end}) }{\left({ V}_{L}\left({t}_{end}\right)+ {V}_{sample}\left({t}_{end}\right)\right)\cdot {t}_{end}}$$

## Supplementary Information

**Additional file 1: Fig. S1**. Cultivation of *G. oxydans* 621H Δ*hsdR* pBBR1p264-FDH-Strep in a RAMOS device with increasing medium component concentration. **Fig. S2**. Extended-batch-cultivation of *G. oxydans* 621H Δ*hsdR* pBBR1p264-FDH-Strep in a 2 L Visual Safety Fermenter (VSF, Bioengineering) with constant feeding of fructose (970 g/L) between 21 and 50 h. **Fig. S3**. Extended-batch-cultivation of *G. oxydans* 621H Δ*hsdR* pBBR1p264-FDH-Strep in a 2 L fermenter (Sartorius) with constant feeding of fructose (1180 g/L) between 18 and 44 h. **Fig. S4**. Correlation of time of lag-phase and 5-ketofructose concentration, osmolality and total sugar concentration. **Fig. S5**. Extended-batch-cultivation of *G. oxydans* 621H Δ*hsdR* pBBR1p264-FDH-Strep in a 50 L pressurised fermenter (Bioengineering) with constant feeding of fructose (1035 g/L) between 13 and 40 h. **Fig. S6**. Extended-batch-cultivation of *G. oxydans* 621H Δ*hsdR* pBBR1p264-FDH-Strep in a 50 L pressurised fermenter (Bioengineering) with constant feeding of fructose (1035 g/L) between 13 and 40 h. **Fig. S7**. Extended-batch-cultivation of *G. oxydans* 621H Δ*hsdR* pBBR1p264-FDH-Strep in a 150 L pressurised fermenter (Frings) with constant feeding of fructose (1035 g/L) between 22 and 52 h. **Fig. S8**. Cultivation of *G. oxydans *621H Δ*hsdR* pBBR1p264-*fdhSCL*-ST in a RAMOS device with 150 g/L fructose. **Tab. S1.** Production of 5-keto-d-fructose.

## Data Availability

The datasets supporting the conclusions of this article are included within the article and the Additional file 1: Figures S1–S8 and Table S1. The datasets used and/or analysed during the current study are available from the corresponding author on reasonable request.

## Abbreviations

5KF: 5-Keto-d-fructose
CTR: Carbon dioxide transfer rate [mmol/L/h]
DHA: Dihydroxyacetone
DOT: Dissolved oxygen tension [% air saturation]
FDH: Fructose dehydrogenase
*G. oxydans fdh*: *G. oxydans* 621H Δ*hsdR* pBBR1-p264-*fdhSCL*-ST
HMF: 5-Hydroxymethylfurfural
HMFCA: 5-Hydroxymethyl-2-furan carboxylic acid
HPLC: High-performance liquid chromatography
OD_600_: Optical density at 600 nm [−]
OTR: Oxygen transfer rate [mmol/L/h]
OTR_max_: Maximum oxygen transfer capacity [mmol/L/h]
RAMOS: Respiration activity MOnitoring System
RQ: Respiratory quotient [−]
vvm: Volumetric aeration rate [gas volume/liquid volume/min]
