# Supplementary material for: Highly efficient fermentation of 5-keto-d-fructose with Gluconobacter oxydans at different scales
Source: Microb Cell Fact. 2022 Dec 10;21:255. doi: 10.1186/s12934-022-01980-5 (PMC9741787; doi:10.1186/s12934-022-01980-5)
Supplement: Supplementary file 1 — Additional file 1: Fig. S1. Cultivation of G. oxydans 621H ΔhsdR pBBR1p264-FDH-Strep in a RAMOS device with increasing medium component concentration. Fig. S2. Extended-batch-cultivation of G. oxydans 621H ΔhsdR pBBR1p264-FDH-Strep in a 2 L Visual Safety Fermenter (VSF, Bioengineering) with constant feeding of fructose (970 g/L) between 21 and 50 h. Fig. S3. Extended-batch-cultivation of G. oxydans 621H ΔhsdR pBBR1p264-FDH-Strep in a 2 L fermenter (Sartorius) with constant feeding of fructose (1180 g/L) between 18 and 44 h. Fig. S4. Correlation of time of lag-phase and 5-ketofructose concentration, osmolality and total sugar concentration. Fig. S5. Extended-batch-cultivation of G. oxydans 621H ΔhsdR pBBR1p264-FDH-Strep in a 50 L pressurised fermenter (Bioengineering) with constant feeding of fructose (1035 g/L) between 13 and 40 h. Fig. S6. Extended-batch-cultivation of G. oxydans 621H ΔhsdR pBBR1p264-FDH-Strep in a 50 L pressurised fermenter (Bioengineering) with constant feeding of fructose (1035 g/L) between 13 and 40 h. Fig. S7. Extended-batch-cultivation of G. oxydans 621H ΔhsdR pBBR1p264-FDH-Strep in a 150 L pressurised fermenter (Frings) with constant feeding of fructose (1035 g/L) between 22 and 52 h. Fig. S8. Cultivation of G. oxydans 621H ΔhsdR pBBR1p264-fdhSCL-ST in a RAMOS device with 150 g/L fructose. Tab. S1. Production of 5-keto-d-fructose. [file 12934_2022_1980_MOESM1_ESM.docx]

# Additional file 1

**Highly efficient fermentation of 5-keto-ᴅ-fructose with *Gluconobacter oxydans* at different scales**

Svenja Battling, Tobias Engel, Elena Herweg, Paul-Joachim Niehoff, Matthias Pesch, Theresa Scholand, Marie Schöpping, Nina Sonntag and Jochen Büchs^*^

AVT-Chair for Biochemical Engineering, RWTH Aachen University, Forckenbeckstraße 51, 52074 Aachen, Germany

*Correspondence: [jochen.buechs@avt.rwth-aachen.de](mailto:jochen.buechs@avt.rwth-aachen.de)

**Fig. S1**. Cultivation of *G. oxydans* 621H Δ*hsdR* pBBR1p264-FDH-Strep in a RAMOS device with increasing medium component concentration.

**Fig. S2**. Extended-batch-cultivation of *G. oxydans* 621H Δ*hsdR* pBBR1p264-FDH-Strep in a 2 L Visual Safety Fermenter (VSF, Bioengineering) with constant feeding of fructose (970 g/L) between 21 and 50 h.

**Fig. S3**. Extended-batch-cultivation of *G. oxydans* 621H Δ*hsdR* pBBR1p264-FDH-Strep in a 2 L fermenter (Sartorius) with constant feeding of fructose (1180 g/L) between 18 and 44 h.

**Fig. S4**. Correlation of time of lag-phase and 5-ketofructose concentration, osmolality and total sugar concentration.

**Fig. S5**. Extended-batch-cultivation of *G. oxydans* 621H Δ*hsdR* pBBR1p264-FDH-Strep in a 50 L pressurised fermenter (Bioengineering) with constant feeding of fructose (1035 g/L) between 13 and 40 h.

**Fig. S6**. Extended-batch-cultivation of *G. oxydans* 621H Δ*hsdR* pBBR1p264-FDH-Strep in a 50 L pressurised fermenter (Bioengineering) with constant feeding of fructose (1035 g/L) between 13 and 40 h.

**Fig. S7**. Extended-batch-cultivation of *G. oxydans* 621H Δ*hsdR* pBBR1p264-FDH-Strep in a 150 L pressurised fermenter (Frings) with constant feeding of fructose (1035 g/L) between 22 and 52 h.

**Fig. S8**. Cultivation of *G. oxydans* 621H Δ*hsdR* pBBR1p264-*fdhSCL*-ST in a RAMOS device with 150 g/L fructose.

**Tab. S1.** Production of 5-keto-ᴅ-fructose.


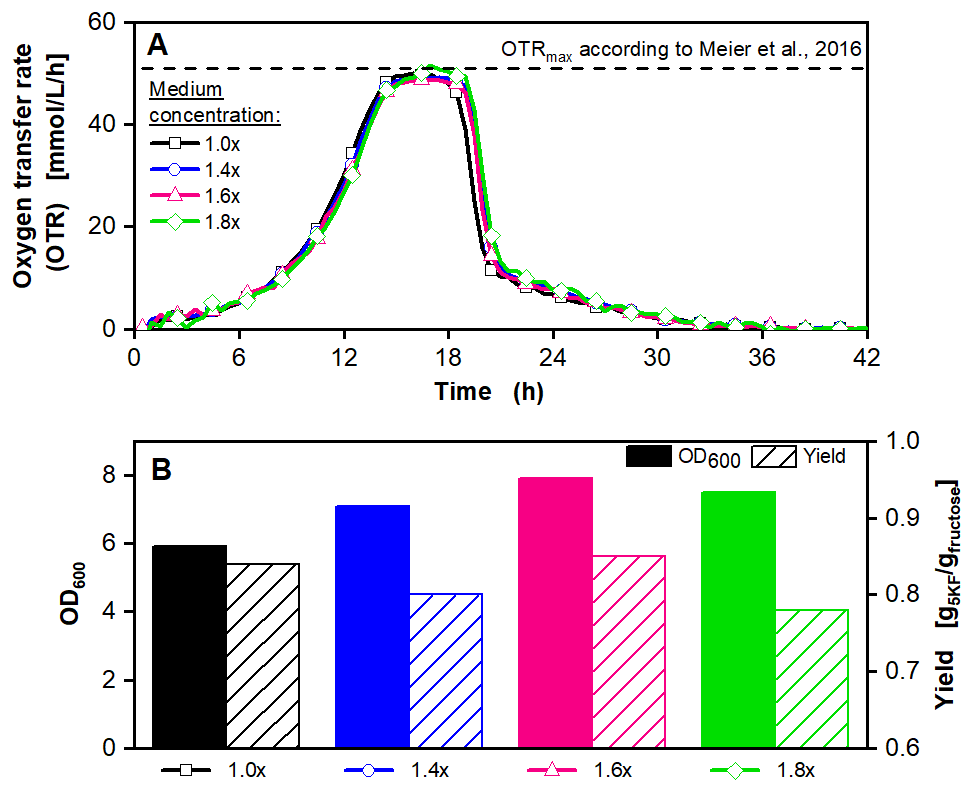


**Fig. S1: Cultivation of *G. oxydans* 621H Δ*hsdR* pBBR1p264-*fdhSCL*-ST in a RAMOS device with increasing medium component concentration.** Depicted is **A** the oxygen transfer rate (OTR) and **B** the optical density OD_600_ (solid bars) and the yield g_5KF_/g_fructose_ (hatched bars). **A**: Maximum oxygen transfer capacity OTR_max_ calculated according to Meier et al., 2016 [45] is displayed as a dashed horizontal line. The cultures are obviously oxygen limited between 15 and 19 h**.** Cultivations were performed in duplicates at 30 °C, 350 rpm, V_L_ = 10 mL in 250 mL shake flasks, initial pH value 6 and a shaking diameter of 50 mm with 150 g/L fructose in complex medium with increasing concentration: 1x concentration (black), 1,2x concentration (blue), 1.6x concentration (pink) and 1.8x concentration (green). Shown are mean values of duplicates.


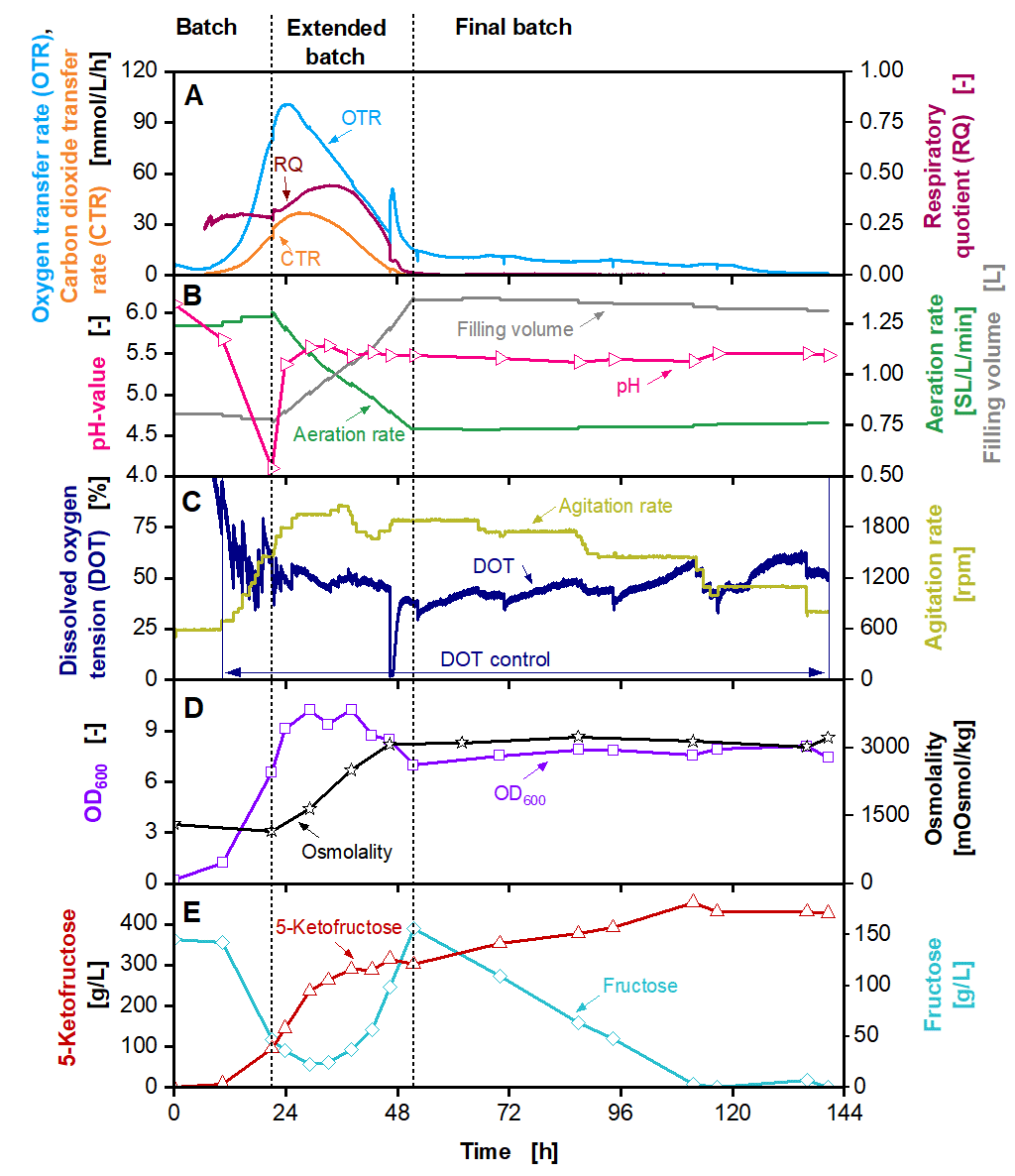


**Fig. S2: Extended-batch-cultivation of *G. oxydans* 621H Δ*hsdR* pBBR1p264-*fdhSCL* -ST in a 2 L Visual Safety Fermenter (VSF, Bioengineering) with constant feeding of fructose (970 g/L) between 21 and 50 h.** Depicted is **A** the oxygen transfer rate (OTR, light blue), carbon dioxide transfer rate (CTR, orange) and respiratory quotient (RQ, dark red), **B** pH (pink), aeration rate (green) and filling volume (grey), **C** the dissolved oxygen tension (DOT, dark blue) and agitation rate (light green), **D** the optical density OD_600_ (purple) and osmolality (black), **E** fructose (light blue) and 5-ketofructose concentration (red). Cultivation was performed in complex medium (concentrated 1.6x) with initial 150 g/L fructose at 30 °C, initial pH value 6, pH control at 5.5 from 21 h with 3 M KOH, V_L,start_ = 0.8 L in a 2 L fermenter. DOT was kept ≥ 30% by variation of agitation speed (500 – 2050 rpm), absolute aeration rate Q_g_ = 1 SL/min. Feeding solution: 970 $\pm$ 5 g_fructose_/L, heat pretreatment: 121 °C, 21 min. Feed rate: 18.8 g_fructose_/h. Fructose feeding solution and peripheral feeding system were heated to ~ 55 °C. After 48 h, a few mL of isopropyl alcohol leaked into the fermenter, which led to the OTR peak at 48 h. RQ-values are only shown, when OTR-values are above 5 mmol/L/h.


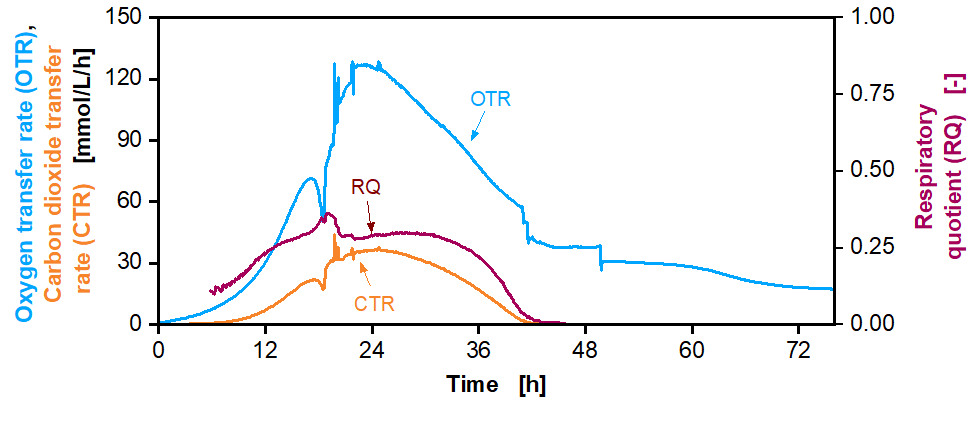


**Fig. S3: Extended-batch-cultivation of *G. oxydans* 621H Δ*hsdR* pBBR1p264-*fdhSCL* -ST in a 2 L fermenter (Sartorius) with constant feeding of fructose (1180 g/L) between 18 and 44 h.** Depicted is the oxygen transfer rate (OTR, light blue), carbon dioxide transfer rate (CTR, orange) and respiratory quotient (RQ, dark red). Cultivation was performed in complex medium (concentrated 1.6x) with 150 g/L initial fructose at 30 °C, initial pH value 6, pH control at 5 from 18 h with 3 M KOH, V_L,start_ = 1 L in a 2 L fermenter. DOT was kept ≥ 30% by variation of agitation speed (500 - 1500 rpm), absolute aeration rate Q_g_ = 1 - 2.5 SL/min. Feeding solution: 1180 $\pm$ 2 g_fructose_/L, heat pretreatment: 100 °C, 10 min. Feed rate: 26.3 g_fructose_/h. Fructose feeding solution and peripheral feeding system were heated to ~ 55 °C. RQ-values are only shown, when OTR-values are above 5 mmol/L/h. Corrected data are shown in Fig. 4.


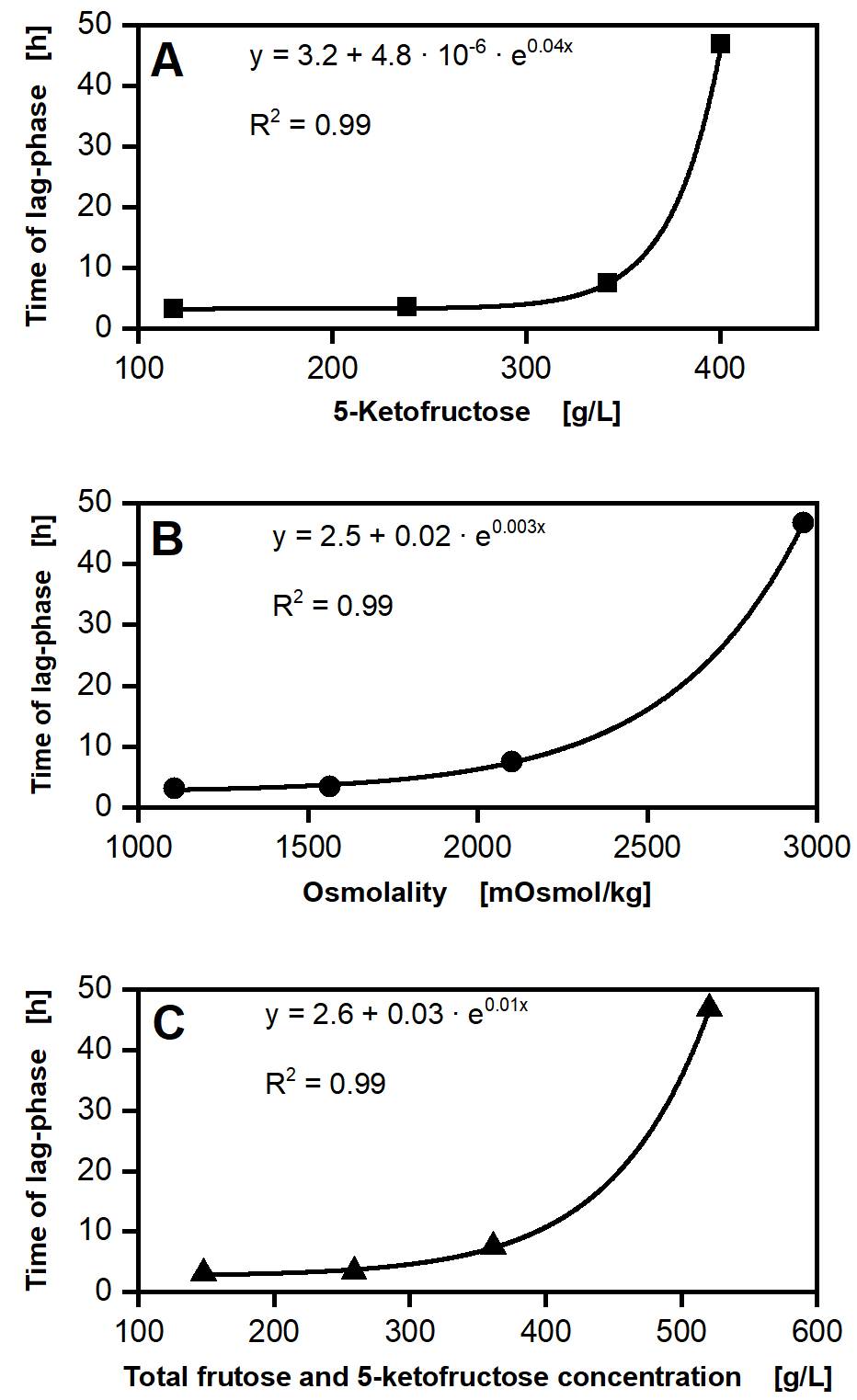


**Fig. S4: Correlation of time of lag-phase and 5-ketofructose concentration, osmolality and total sugar concentration.** Determination of lag phases from cultivations displayed in Fig. **5** according to Palmen et al., 2013, correlated with the **A** 5-ketofructose concentration, **B** osmolality and **C** total fructose and 5-ketofructose concentrations at timepoints, where samples were taken during an extended batch fermentation (Fig. 4). Solid lines represent exponential fit. Equation and standard deviations are given in the graphs.


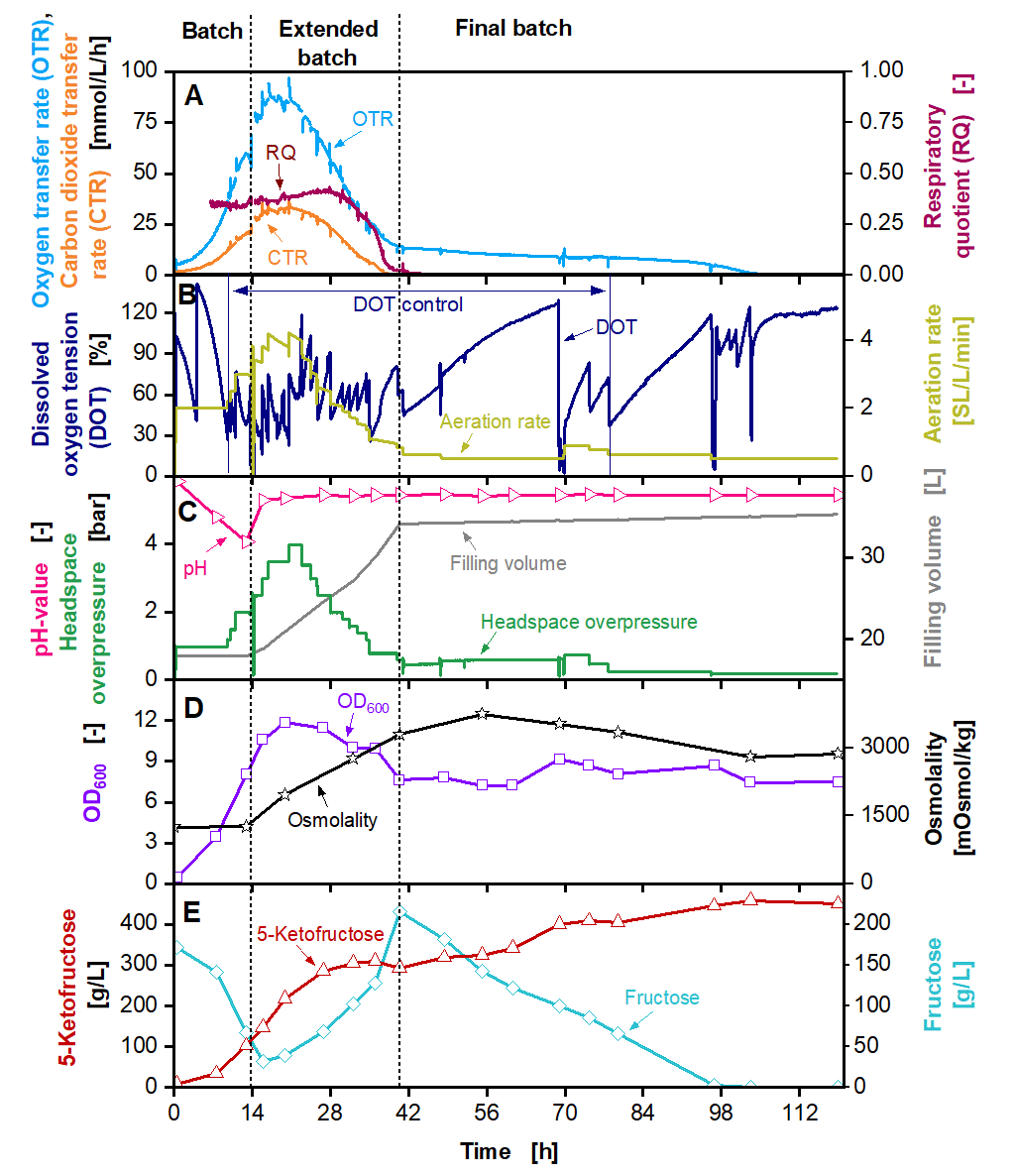


**Fig. S5: Extended-batch-cultivation of *G. oxydans* 621H Δ*hsdR* pBBR1p264-*fdhSCL* -ST in a 50 L pressurised fermenter (Bioengineering) with constant feeding of fructose (1035 g/L) between 13 and 40 h.** Depicted is **A** the oxygen transfer rate (OTR, light blue), carbon dioxide transfer rate (CTR, orange) and respiratory quotient (RQ, dark red), **B** the dissolved oxygen tension (DOT, dark blue) and aeration rate (light green), **C** pH (pink), headspace overpressure (green) and filling volume (grey), **D** the optical density OD_600_ (purple) and osmolality (black), **E** fructose (light blue) and 5-ketofructose concentration (red). Cultivation was performed in complex medium (concentrated 1.6x) with 170 g/L initial fructose at 30 °C, initial pH value 6, pH control at 5 from 13 h with 3 M KOH, V_L,start_ = 18 L in a 50 L pressurised fermenter (Bioengineering). Initial OD_600_ value 0.5. DOT was kept ≥ 30% by variation of headspace overpressure (0.2 - 4 bar), agitation rate: 500 rpm, absolute aeration rate Q_g_ = 18 - 90 SL/min was increased linearly in parallel to the headspace overpressure. Feeding solution: 1035 $\pm$ 30 g_fructose_/L, heat pretreatment: 100 °C, 20 min. Feed rate: 540 g_fructose_/h. Fructose feeding solution and peripheral feeding system were heated to ~ 55 °C. RQ-values are only shown, when OTR-values are above 5 mmol/L/h. For clarity, noisy data were deleted. For raw data please refer to Fig. S6.


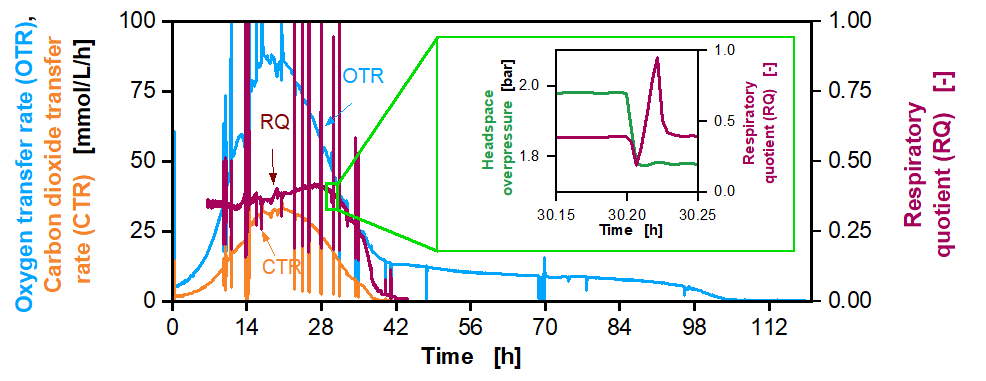


**Fig. S6: Extended-batch-cultivation of *G. oxydans* 621H Δ*hsdR* pBBR1p264-*fdhSCL* -ST in a 50 L pressurised fermenter (Bioengineering) with constant feeding of fructose (1035 g/L) between 13 and 40 h.** Raw data of Fig. S5. Depicted is the oxygen transfer rate (OTR, light blue), carbon dioxide transfer rate (CTR, orange) and respiratory quotient (RQ, dark red). The green inlay shows RQ and headspace overpressure from 30.15 – 30.25 h, illustrating the origin of the noisy data. Cultivation was performed in complex medium (concentrated 1.6x) with 170 g/L initial fructose at 30 °C, initial pH value 6, pH control at 5 from 13 h with 3 M KOH, V_L,start_ = 18 L in a 50 L pressurised fermenter (Bioengineering). Initial OD_600_ value 0.5. DOT was kept ≥ 30% by variation of headspace overpressure (1 - 1.8 bar), agitation rate: 500 rpm, absolute aeration rate Q_g_ = 18 - 90 SL/min increased proportionally to headspace overpressure. Feeding solution: 1035 $\pm$ 30 g_fructose_/L, heat pretreatment: 100 °C, 20 min. Feed rate: 540 g_fructose_/h. Fructose feeding solution and peripheral feeding system were heated to ~ 55 °C. RQ-values are only shown, when OTR-values are above 5 mmol/L/h.


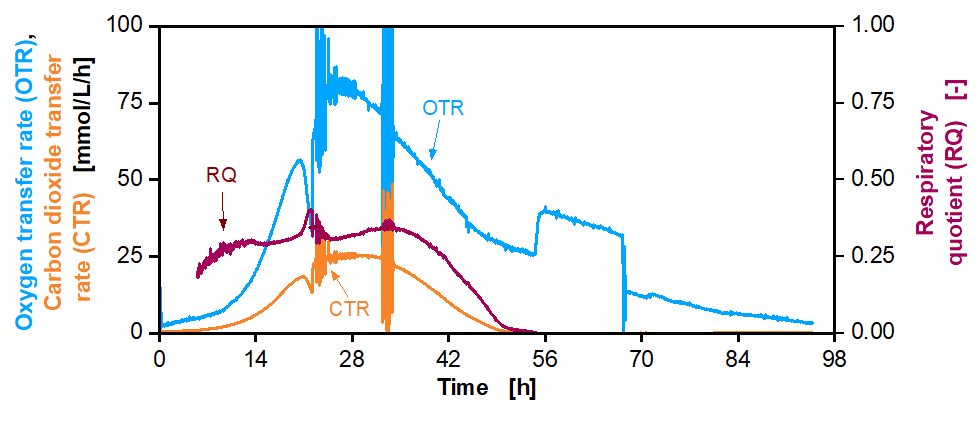


**Fig. S7: Extended-batch-cultivation of *G. oxydans* 621H Δ*hsdR* pBBR1p264-*fdhSCL* -ST in a 150 L pressurised fermenter (Frings) with constant feeding of fructose (1035 g/L) between 22 and 52 h.** Raw data of Fig. 7. Depicted is the oxygen transfer rate (OTR, light blue), carbon dioxide transfer rate (CTR, orange) and respiratory quotient (RQ, dark red). Cultivation was performed in complex medium (concentrated 1.6x) with 150 g/L initial fructose at 30 °C, initial pH value 6, pH control at 5 from 22 h with 3 M KOH, V_L,start_ = 50 L in a 150 L pressurised fermenter (Frings). DOT was kept ≥ 30% by variation of headspace overpressure (1 - 1.8 bar), agitation rate: 600 rpm, absolute aeration rate Q_g_ = 70 - 170 SL/min was increased linearly in parallel to the headspace overpressure. Feeding solution: 745 $\pm$ 25 g_fructose_/L, heat pretreatment: 100 °C, 4 h. Feed rate: 900 g_fructose_/h. Fructose feeding solution was heated to ~ 50 °C. RQ-values are only shown, when OTR-values are above 5 mmol/L/h. Technical problems occurring during the fermentation between 22 and 26 h: DOT controller parameters for aeration rate and headspace pressure were not correctly implemented; between 32 and 35 h: for aeration rates below and above 2 SL/L/min two different mass flow controllers were used, at 2 SL/L/min switching between mass flow controllers was not correctly implemented; between 54 and 66 h: gas flow rate from the fermenter into off gas analyser was too low.


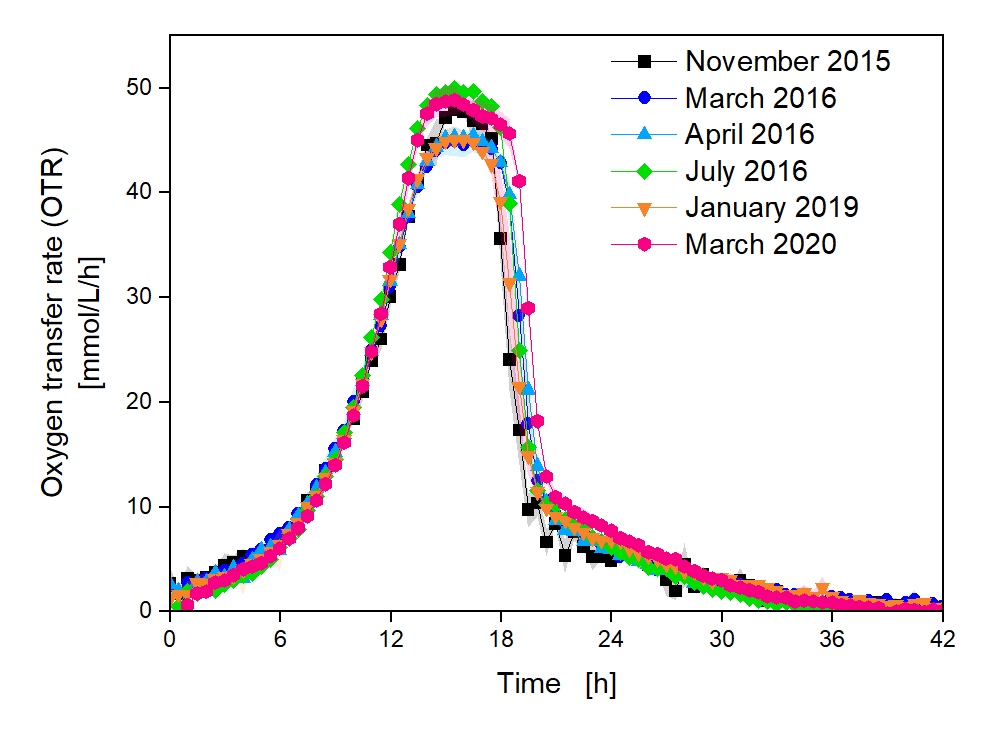


**Fig. S8: Cultivation of *G. oxydans* 621H Δ*hsdR* pBBR1p264-*fdhSCL*-ST in a RAMOS device with 150 g/L fructose.** Depicted is the oxygen transfer rate (OTR). Lag phases were adjusted by a maximum of 5 h, to allow an easier comparison of OTR curves. Reference cultivations were performed over a period of more than 4 years using same cultivation conditions. Cultivations were performed in duplicates in 1.0x concentrated medium at 30 °C, 350 rpm, V_L_ = 10 mL in 250 mL shake flasks, initial pH value 6 and a shaking diameter of 50 mm Shown are mean values of duplicates. The shadows around the curves indicate the highest and lowest values. The shadows are hardly visible because of the small deviation.

**Tab. S1: Production of 5-keto-ᴅ-fructose.**

| **Fig./ Reference** | **Experiment** | **Fermenter** | **Total fructose mass** | **Total 5KF mass** | **Titre** | **Yield** | **Productivity** |
| --- | --- | --- | --- | --- | --- | --- | --- |
|  |  |  | **[g]** | **[g]** | **[g_5KF_/L]** | **[g_5KF_/g_fructose_]** | **[g/L/h]** |
| Fig. 1 | Extended batch (1.0x conc. medium) with feeding of 1035 g/L fructose | 2 L (VSF, Bioengineering) | 645 | 591 | 462 | 0.92 | 3.0 |
| Fig. S2 | Extended batch (1.6x conc.medium) with feeding of 970 g/L fructose | 2 L (VSF, Bioengineering) | 735 | 641 | 454 | 0.87 | 4.1 |
| Fig. 4 | Extended batch (1.6x conc.medium) with feeding of 1180 g/L fructose | 2 L (Sartorius) | 861 | 848 | 545 | 0.98 | 7.6 |
| Fig. 6 | Extended batch (1.6x conc. medium) with solid fructose feeding | 2 L (Sartorius) | 798  $\pm$ 72 | 707 | 511 | 0.89  $\pm$ 0.09 | 8.0 |
| Fig. S5 | Extended batch (1.6x conc. medium) with feeding of 1035 g/L fructose | 50 L pressurised fermenter (Bioengineering) | 18,436 | 15,713 | 460 | 0.85 | 4.5 |
| Fig. 7 | Extended batch (1.6x conc.medium) with feeding of 745 g/L fructose | 150 L pressurised fermenter (Frings) | 37,336 | 36,510 | 385 | 0.98 | 4.1 |
| [14] | Extended batch (1.0x conc.medium) with feeding of 1035 g/L fructose | 2 L (Sartorius) | 980 | 904 | 489 | 0.92 | 7.0 |
